# Evaluation of the Relationships Between Microbiota and Metabolites in Soft-Type Ripened Cheese Using an Integrated Omics Approach

**DOI:** 10.3389/fmicb.2021.681185

**Published:** 2021-06-08

**Authors:** Ryosuke Unno, Toshihiro Suzuki, Minenosuke Matsutani, Morio Ishikawa

**Affiliations:** ^1^Department of Fermentation Science, Faculty of Applied Bioscience, Tokyo University of Agriculture, Tokyo, Japan; ^2^NODAI Genome Research Center, Tokyo University of Agriculture, Tokyo, Japan

**Keywords:** cheese, metagenomics, microbiota, metabolome, marine bacteria, correlation analysis

## Abstract

Cheese ripening is effected by various microorganisms and results in the characteristic flavors of cheese. Owing to the complexity of the microbiota involved, the relationship between microorganisms and components during ripening remains unclear. In this study, metagenomics and metabolomics were integrated to reveal these relationships in three kinds of surface mold-ripened cheeses and two kinds of bacterial smear-ripened cheeses. The microbiota is broadly divided into two groups to correspond with different cheese types. Furthermore, surface mold-ripened cheese showed similar microbiota regardless of the cheese variety, whereas bacterial smear-ripened cheese showed specific microbiota characterized by marine bacteria (MB) and halophilic and alkaliphilic lactic acid bacteria for each cheese variety. In the metabolite analysis, volatile compounds suggested differences in cheese types, although organic acids and free amino acids could not determine the cheese characteristics. On the other hand, Spearman correlation analysis revealed that the abundance of specific bacteria was related to the formation of specific organic acids, free amino acids, and volatile compounds. In particular, MB was positively correlated with esters and pyrazines, indicating their contribution to cheese quality. These methodologies and results further our understanding of microorganisms and allow us to select useful strains for cheese ripening.

## Introduction

Cheese is one of the most common fermented foods in the world and has many varieties. In general, cheesemaking involves several processes, the coagulation of milk, separating whey from curd, salting, and ripening. In detail, these process are different depending on the cheese varieties (Almena-Aliste and Mietton, 2014). For example, three methods are used for milk coagulation as follows: acid, which is derived from starter lactic acid bacteria (SLAB), rennet, and acid/heat (McSweeney et al., 2017). In draining the whey, there are various methods such as cutting, cooking, stirring, and pressing (Kindstedt, 2014). Among these, ripening is a crucial process that is responsible for the characteristic flavor, texture, and appearance of each cheese type (Fox and McSweeney, 2017). Examples of how differences in ripening affect the characteristics include surface mold-ripened cheeses, such as Brie de Meaux, and bacterial smear-ripened cheese, such as Maroilles. The flavor and appearance of these two types of cheeses are significantly different after ripening, although they are classified as soft uncooked unpressed cheeses, which are similar in the manufacturing process from milk to curd (Almena-Aliste and Mietton, 2014). To make surface mold-ripened cheese, a suspension of *Penicillium camemberti* is sprayed onto the surface before ripening at low temperatures (11–13°C) for at least 21 days (Kindstedt, 2014; Spinnler, 2017). In contrast, bacterial smear-ripened cheese are washed with brine every few days during ripening under cold conditions (4–20°C) for up to 63 days (Kindstedt, 2014; Mounier et al., 2017). At the beginning of ripening, the pH value is typically approximately 5 because of acidification by SLAB (Monnet et al., 2015). When ripening starts, a variety of yeast and *P. camemberti* is developed on the surface of mold-ripened cheese, whereas halotolerant yeasts are developed on bacterial smear-ripened cheese. In general, it is known that these fungi raise the pH by metabolizing lactate to CO_2_ and H_2_O oxidatively and producing alkaline metabolites such as NH_3_ from amino acids in cheeses (McSweeney, 2004; Gori et al., 2007; Fröhlich-Wyder et al., 2019). The pH at the cheese surface can reach a value higher than 7.5 at the end of ripening, allowing not only starters but also non-starters, such as acid-sensitive bacteria, to grow (Monnet et al., 2015). Some non-starter bacteria and fungi have been shown to play a role in cheese ripening. For example, non-starter lactic acid bacteria (NSLAB), such as hetero-fermentative *Lactobacillus*, *Pediococcus*, *Enterococcus*, *Leuconostoc*, and *Carnobacterium*, establish themselves in ripened cheese from raw milk, cheesemaking environments, and natural starter cultures, and contribute to the final characteristics of cheese (Afzal et al., 2010; Settanni and Moschetti, 2010; Pogačić et al., 2016; Gobbetti et al., 2018; Eugster et al., 2019; D’Angelo et al., 2020). On the other hand, *Brevibacterium* has been recognized as a ubiquitous microorganism in bacterial smear-ripened cheese and plays an important role in the development of characteristic orange color and flavor (Rattray and Fox, 1999; Anast et al., 2019). As for fungi, *Geotrichum candidum* and *Debaryomyces hansenii* are commonly found in ripened cheeses and contribute to cheese flavor and texture because of their deacidifying, proteolytic, and/or lipolytic activity (McSweeney, 2004; Irlinger et al., 2015; Fröhlich-Wyder et al., 2019). Thus, various microorganisms present during the ripening period affect the cheese characteristics, especially with respect to the formation of flavor. Moreover, specific microbiota in each cheese is considered to be caused by a difference in ripening methods. Traditionally, this secondary microbiota that develop during ripening is adventitious and is acquired from the milk and/or environment. Since this method is likely to bring about variable microbiota, inconsistencies in cheese quality often occur (Fox et al., 2017). Therefore, in modern cheese technology, the adventitious microbiota are selected as adjunct cultures and are artificially added during cheesemaking (Fox et al., 2017). However, the role of some microorganisms that adventitiously grow during cheese ripening is still unclear. Furthermore, due to the diversity and complexity of microbiota and components that constitute ripened cheese, the overall relationship between flavor development and existing microorganisms during ripening remains unclear.

Recently, metagenomics approach that employed high-throughput sequencing technologies revealed the microbiota of various ripened cheeses in detail. Further, it has been revealed that various bacteria such as *Actinobacteria*, *Proteobacteria*, and halophilic and alkaliphilic lactic acid bacteria (HALAB) that did not originate from starter and artificial secondary cultures were found to dominate the cheese microbiota (Irlinger et al., 2015; Dugat-Bony et al., 2016). Furthermore, the application of metabolomics enables the comprehensive analysis of the components of ripened cheese, including the characteristic metabolites (Ochi et al., 2012; Pisano et al., 2016). Over the past few years, the integration of meta-omics technologies has gained attention (Chen et al., 2017; Afshari et al., 2020a). In fermented foods, such as table olives and fermented dairy beverages, metagenomic and metabolomic analyses have revealed a correlation between microorganisms and metabolites (Walsh et al., 2016; Randazzo et al., 2017; Wurihan et al., 2019). These methods could be powerful tools to provide comprehensive and in-depth insight into the complex associations between microbiota and components in ripened cheese. The obtained data by applying this approach will contribute to elucidating the role of non-starter microorganisms and to facilitating targeting to screen microorganisms that are candidates for adjunct culture. Therefore, this information could provide new insights into efficient and sustainable cheesemaking and control cheese quality. Nevertheless, integrating metagenomics and metabolomics approach has not been applied to the verification of cheese, except for that of cheddar cheese (Afshari et al., 2020b).

Therefore, an integrated approach combining metagenomics and metabolomics were applied to reveal the comprehensive relationships between microorganisms and components in the two different types of soft uncooked unpressed cheeses, surface mold-ripened cheese, and bacterial smear-ripened cheese, which are similar in the manufacturing process from milk to curd but differ in the ripening processes. In this study, the microbiota and metabolites were analyzed using metagenomic amplicon sequencing, high-performance liquid chromatography (HPLC), and headspace gas chromatography mass spectrometry (HS-GC/MS).

## Materials and Methods

### Cheese Samples

Three types of surface mold-ripened cheeses (Brie de Meaux, Brie de Melun, and Coulommiers) and two kinds of bacterial smear-ripened cheeses (Langres and Maroilles) were used in this study. Each type of cheese was purchased three times in the food market in Tokyo at different times. After dividing these cheeses into rind and core, a total of 30 samples (e.g., five kinds of cheeses, *n* = 3, and rind and core) were used for the metagenomic sequencing analysis, and to analyze various organic acids, free amino acids, and volatile compounds. The NaCl concentration and pH were determined using a C-121 salinity meter (HORIBA, Ltd., Kyoto, Japan) and an HM-25G pH meter (DKK-TOA Corporation, Tokyo, Japan), as described previously (Unno et al., 2020).

### Metagenomic Sequencing

Total DNA was extracted from cheese samples using the Quick-DNA^TM^ Fecal/Soil Microbe Miniprep Kit (Zymo Research, Irvine, CA, United States) with some modifications. Cheese samples (150 mg) from the rind or core were homogenized in 750 μL Bashing Bead Buffer using BioMasher SP (Nippi, Tokyo, Japan). After transferring the suspension to a ZR BashingBead^TM^ Lysis Tube, DNA was extracted according to the manufacturer’s instructions. The extracted DNA was quantified using the Qubit dsDNA BR Assay Kit (Invitrogen, Carlsbad, CA, United States). 16S rRNA and ITS2 gene amplicon libraries were prepared following the Illumina protocol (Illumina Inc, 2013). For bacteria, the V3 and V4 regions of the 16S rRNA gene were amplified using the forward primer (5′-CCTACGGGNGGCWGCAG-3′) and the reverse primer (5′-GACTACHVGGGTATCTAATCC-3′) with an overhang adapter (Klindworth et al., 2013). For fungi, the ITS2 regions were amplified using the forward primer (5′-GCATCGATGAAGAACGCAGC-3′) and the reverse primer (5′-TCCTCCGCTTWTTGWTWTGC-3′) with an overhang adapter (White et al., 1990; Toju et al., 2012). After amplification, dual index and Illumina sequence adapters were added to the amplified products using the Nextera XT Index Kit (Illumina, San Diego, CA, United States). PCR products were purified with Agencourt AMPure XP (Beckman Coulter, Brea, CA, United States) and read size was checked using an Agilent 2200 Tape Station (Agilent, Santa Clara, CA, United States). The libraries were diluted to 5 nM with 10 mM Tris (pH 8.5) based on qPCR results using the Kapa Library Quantification Kit (Kapa Biosystems, Wilmington, MA, United States), and 2 × 300 bp paired-end sequences were performed using the MiSeq Reagent Kit v3 on an Illumina MiSeq sequencing platform (Illumina, San Diego, CA, United States).

### Assign Taxonomy by QIIME2

The QIIME2 pipeline was used for quality control and taxonomic classification (Bolyen et al., 2019). Sequence reads were imported into QIIME2 and DADA2 (Callahan et al., 2016) to perform denoising and merging to generate amplicon sequence variants (ASVs). The bacterial sequences were subjected to paired-end read analysis. Regarding fungi, the sequence reads were processed with single-end reads using only the forward reads because of insufficient read overlap. With the obtained ASV as a query sequence the bacterial and fungal taxonomy was assigned using Naive Bayes classifier pre-trained on Silva 16S rRNA gene database 132 (Quast et al., 2013) and UNITE database version 8.0 (Nilsson et al., 2019), respectively.

### Organic Acids and Free Amino Acids Analysis

One gram of cheese sample was homogenized in 2 mL ultrapure water with BioMasher SP (Nippi, Tokyo, Japan) and centrifuged for 5 min at 20,000 × *g* at 20°C. The solution was treated with a twofold volume of 5% trichloroacetic acid solution for deproteinization and centrifuged for 10 min at 20,000 × *g* at 20°C. The supernatant was filtered through an Ultrafree^®^-MC Centrifugal Filter Unit (pore size, 0.2 μm; EDM Millipore, Billerica, MA, United States) by centrifuging for 5 min at 16,000 × *g* at 20°C prior to analysis. Organic acids and free amino acids were analyzed using HPLC, as previously described (Suzuki et al., 2021).

### Volatile Compounds Analysis

Volatile compounds were determined using HS-GC/MS using a GCMS-TQ8040 NX trap system (Shimadzu Corporation, Kyoto, Japan) with an Agilent J&W GC-DB-WAX column (Agilent, Santa Clara, CA, United States). Samples (0.5 g) were placed in TORAST HS vials (Shimadzu GLC, Tokyo, Japan), and the temperature program was executed by agitating the vials at 50°C for 30 min. Subsequently, the temperature was maintained at 50°C for 5 min, increased to 250°C at a rate of 10°C/min, and then kept at 250°C for 10 min. The mass spectrometry range was set between 33 and 400 *m/z* in the scan mode. Peak identification was performed by a similarity search of the NIST17 mass spectral library^1^ and retention time comparison. In this study, the peaks detected from the 1.5–20 min were adopted for the analysis.

### Statistical Analysis

All statistical analyses and graphical plotting were performed using *R* (R Core Team, 2019). Principal component analysis (PCA) and correspondence analysis (CA) were performed using the *ropls* package (Thévenot et al., 2015) and the *ca* package (Nenadić and Greenacre, 2007), respectively. Biplots were generated using the *plot* function. Correlation analysis was performed for the sequence data and metabolomics data. Correlation was computed using the *cor* function (method = “spearman”) and tested for *p*-value using the *cor.test* function (*p*-value < 0.05, regarded as statistically significant). Heat maps and dendrograms were generated using the *gplots* package (Warnes et al., 2020).

## Results

### Salinity and pH of Sample Cheeses

The salinity and pH of five kinds of cheese samples were confirmed. Each sample was divided into the rind and core before measurements. The NaCl concentration of rind (2.0–3.0%) was slightly higher than that of the core (1.0–2.0%) in all samples. The pH of rind (6.7–8.3) was higher than that of the core (5.0–7.4). Overall, the rind can be regarded as a slightly alkaline environment with a pH of approximately 8.0, except for Langres samples. The obtained data are shown in Table 1.

**TABLE 1:** The salinity and pH of five kinds of cheese samples.

Sample	NaCl (%)		pH	
	Core	Rind	Core	Rind
Brie de Meaux	2.0	2.0–3.0	6.6–6.8	7.8–8.0
Brie de Melun	2.0	3.0	7.3–7.4	7.5–7.6
Coulommiers	2.0	2.0–3.0	5.6–6.5	7.6–7.8
Langres	1.0	2.0	5.0–5.2	6.7–6.8
Maroilles	2.0	2.0	5.9–6.0	8.2–8.3

*Each sample was divided into rind and core before measurement.*

### Identification of Bacteria Dominating Ripened Cheeses

**FIGURE 1:**
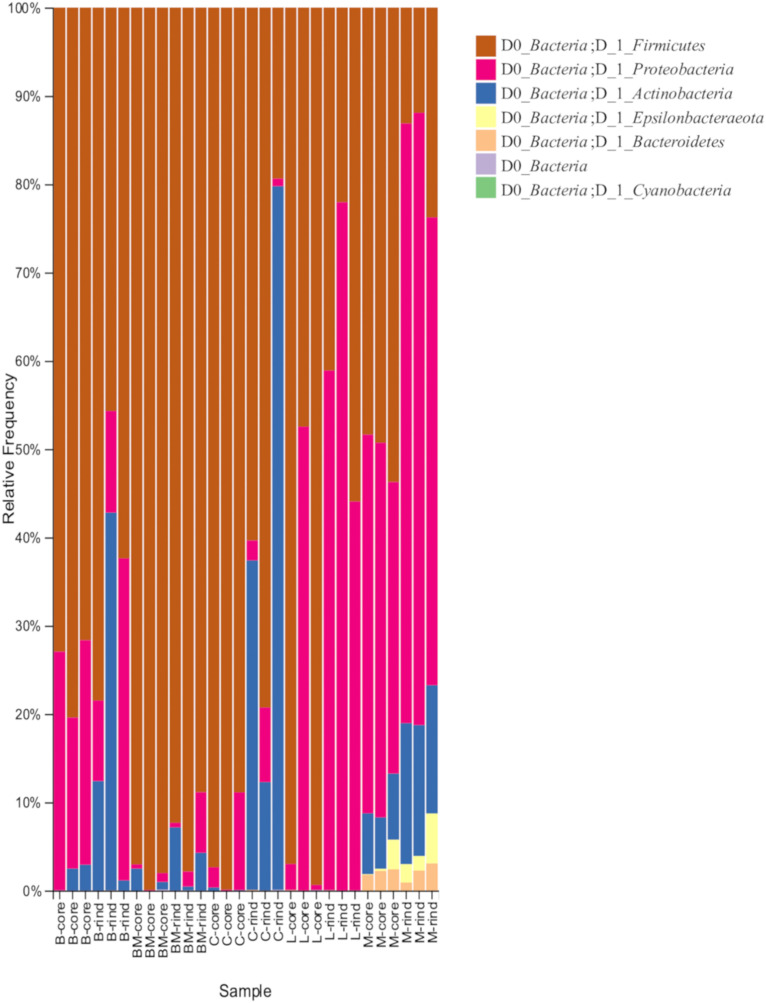
Relative abundance of bacteria phylum in 30 cheese samples. B, Brie de Meaux; BM, Brie de Melun; C, Coulommiers; L, Langres; M, Maroilles.

When 30 cheese samples were subjected to 16S rRNA gene V3-V4 amplicon sequencing analysis, 7,856,332 reads were obtained. Denoising yielded 3,350,955 reads, from which 579 ASVs were identified. To verify the bacterial microbiota between the two types of cheese with different ripening methods (surface mold-ripened cheese and bacterial smear-ripened cheese), ASVs generated from 48,953 reads per sample were identified at the phylum level (Figure 1). All cheese samples were dominated by *Firmicutes*, *Proteobacteria*, and *Actinobacteria*, or some of them. Among these, bacterial smear-ripened cheese had a higher proportion of *Proteobacteria* than surface mold ripened cheese. Langres hardly had *Actinobacteria*. Maroilles also had other phyla, including *Epsilonbacteraeota* and *Bacteroidetes*, along with the above-mentioned phyla.

**FIGURE 2:**
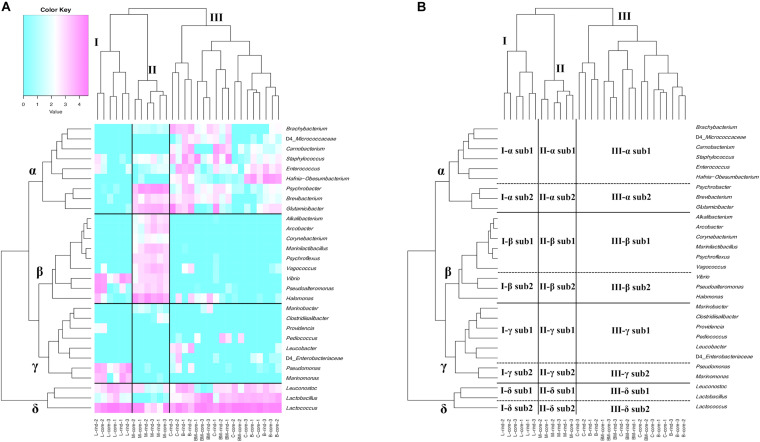
Hierarchical clustered heat map based on the relative abundance of bacteria genus detected on 30 cheese samples. **(A)** Relative abundance of bacteria genus is shown on heat map with dendrogram. Only bacteria with relative abundance > 0.5% in at least one sample are shown. Purple indicates high occupancy and light blue indicates low or non-existent occupancy. **(B)** Hierarchically clustered map. Cheese sample clusters are represented by I, II, and III. Bacteria clusters are represented by α, β, γ, and δ. Each bacteria cluster is further divided into sub1 and sub2. B, Brie de Meaux; BM, Brie de Melun; C, Coulommiers; L, Langres; M, Maroilles.

At the genus level, 76 bacteria were identified using ASVs. Among them, 29 bacteria were present with a relative abundance > 0.5% in at least one sample. Hierarchical cluster analysis based on relative abundance at the genus level showed that cheese samples were separated into characteristic clusters corresponding to the existence of specific bacterial communities (Figure 2). The cheese samples were divided into three major clusters (I, II, and III). Clusters I and II were composed of Langres and Maroilles samples, respectively, which belong to bacterial smear-ripened cheese. Cluster III was composed of surface mold-ripened cheese samples. Within each cluster, the rind and core microbiota coexist. Similarly, bacterial communities were distributed into four major clusters (α, β, γ, and δ). Furthermore, each cluster was distributed into two sub-clusters (sub1 and sub2). Cluster α sub1 consisted of *Enterococcus* and *Carnobacterium*, which are known to be NSLAB in addition to *Staphylococcus*, *Brachybacterium*, and *Hafnia-Obesumbacterium*. Cluster α sub2 consisted of *Brevibacterium* and *Glutamicibacter*, which belong to the phylum *Actinobacteria*, and *Psychrobacter*, which is considered a marine-originated *Proteobacteria* designated as marine bacteria (MB). Cluster β sub1 consisted of *Marinilactibacillus*, *Alkalibacterium*, and *Vagococcus* (HALAB), *Corynebacterium* (phylum *Actinobacteria*), *Psychroflexus* (phylum *Bacteroidetes*), and *Arcobacter* (phylum *Epsilonbacteraeota*). Cluster β sub2 consisted of *Halomonas*, *Pseudoalteromonas*, and *Vibrio*, which belong to the MB. In cluster γ, sub1 consisted of *Pediococcus*, which is known to be NSLAB and *Leucobacter* of the phylum *Actinobacteria*, while sub2 consisted of *Marinomonas* and *Pseudomonas*, which were considered as MB. Cluster δ sub1 consisted of *Lactobacillus* and *Leuconostoc*, which are generally used as SLAB, such as *Lactobacillus delbrueckii* and *Leuconostoc mesenteroides*. Cluster δ sub2 was assigned to *Lactococcus*, which is known as SLAB.

Cluster I, composed of Langres samples, formed a characteristic bacterial microbiota consisting of lactic acid bacteria (LAB) (I-δ) and MB (I-β sub2 and I-γ sub2). Cluster II, composed of Maroilles samples, showed a low abundance of cluster δ bacteria, especially δ sub1 (II-δ), compared with the abundance in other cheeses. Additionally, this cluster was dominated by the bacteria belonging to the cluster α sub2 and cluster β bacteria characterized by the existence of MB, HALAB, and *Actinobacteria* (II-α sub2 and II-β). Cluster III, composed of surface mold-ripened cheese samples, was widely occupied by the bacteria belonging to the cluster α and δ (III-α and III-δ). Furthermore, *Leucobacter* was detected in Brie de Meaux and Coulommiers, while *Pediococcus* was detected in Brie de Melun (III-γ).

Thus, surface mold-ripened cheese formed large clusters regardless of cheese varieties, whereas bacterial smear-ripened cheese also formed large clusters, which were further divided by cheese variety. Moreover, it was indicated that MB and HALAB are characteristic constituents, especially in bacterial smear-ripened cheese microbiota.

### Identification of Fungi Dominating Ripened Cheeses

Fungi were also identified using the ITS2 region based on the same cheese samples. ITS amplicon sequencing analysis on 30 cheese samples yielded 9,039,163 reads; of these, 2,385,721 reads remained after denoising. Among the 29 fungi that were identified at the genus level from 20,000 reads per sample, 10 fungi were present with relative abundance > 0.5% in at least one sample (Supplementary Figure 1).

Cheese samples were distributed into four major clusters (I, II, III, and IV) by hierarchical cluster analysis based on relative abundance at the genus level. Cluster I consisted of the Maroille samples. Cluster II consisted of Langres samples. Cluster III mainly consisted of Brie de Melun samples. Cluster IV consisted of Brie de Meaux and Coulommiers samples. Similarly, fungal communities were distributed into four major clusters (α, β, γ, and δ). Furthermore, cluster α was distributed into two sub-clusters (sub1 and sub2). Cluster α sub1 and cluster α sub2 were assigned to *Dipodascus* and *Penicillium*, respectively. Cluster β was assigned to *Debaryomyces*. Cluster γ consisted of *Kluyveromyces*, *Candida*, *Saturnispora*, *Pichia*, and *Cyberlindnera*. Cluster δ was assigned to *Scopulariopsis*.

Cluster I consisted of Maroilles samples that were strongly dominated by *Debaryomyces* alone (I-β). Cluster II, consisting of Langres samples, was dominated by *Dipodascus* in addition to *Debaryomyces* (II-α sub1 and II-β). Further, clusters III and IV were dominated by *Dipodascus* and *Penicillium* (III-α and IV-α). Additionally, *Scopulariopsis* was detected in cluster III and *Candida* and *Kluyveromyces* in cluster IV (III-δ and IV-γ). In general, *Geotrichum candidum* is found in cheese. This species is synonymous with *Dipodascus geotrichum*. Although our results have limitations in identification at the species level, it can be considered that ASVs identified as *Dipodascus* were likely to be *G. candidum*. Thus, *Penicillium*, *Debaryomyces*, and *Dipodascus* were the predominant fungal microbiota in the cheese samples. This result suggests that the fungal microbiota is dominated by a small number of species compared with the bacterial microbiota.

### Relationship Between Cheese Variety and Components

**FIGURE 3:**
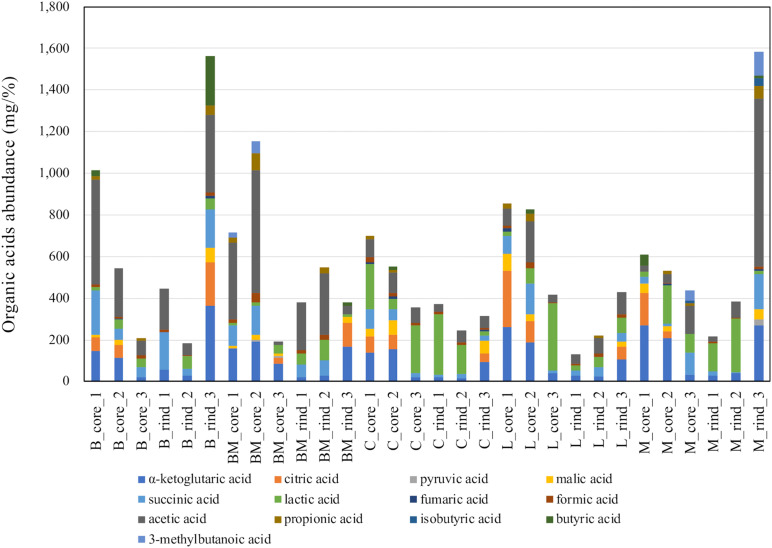
Abundances of organic acids in 30 cheese samples. The total amount of organic acid in each sample is shown in the barplot. B, Brie de Meaux; BM, Brie de Melun; C, Coulommiers; L, Langres; M, Maroilles.

**FIGURE 4:**
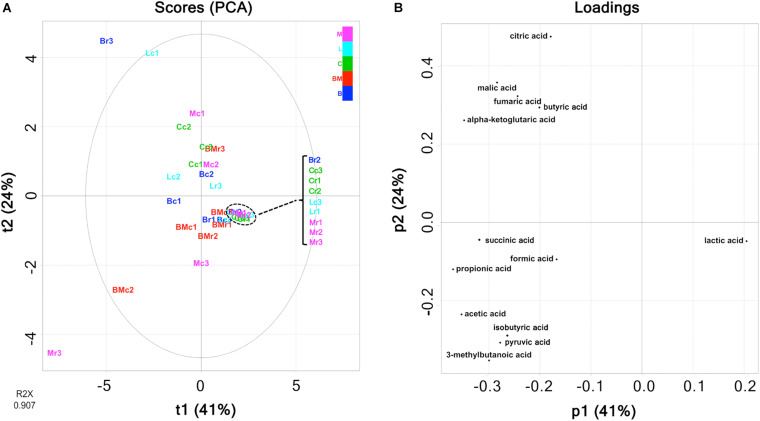
Principal component analysis (PCA) on the abundance of organic acids in 30 cheese samples. **(A)** Score plot. **(B)** Loading plot. Bc, Brie de Meaux core; Br, Brie de Meaux rind; BMc, Brie de Melun core; BMr, Brie de Melun rind; Cc, Coulommiers core; Cr, Coulommiers rind; Lc, Langres core; Lr, Langres rind; Mc, Maroilles core; Mr, Maroilles rind.

Multivariate analysis of metabolites (organic acids, free amino acids, and volatile compounds) was performed to reveal the characteristic components of each ripened cheese. Organic acids were quantified using HPLC (Supplementary Table 1), and the amounts are summarized in Figure 3. The tendency of acetate accumulation in Brie de Meaux and Brie de Melun, as well as that of lactate accumulation in Coulommiers was observed. However, overall, the amount of each organic acid was variable for each cheese sample. Therefore, PCA was performed in an attempted to reveal the characteristics of organic acid contents in each cheese variety. The PCA score and loading plots of the first two principal components of the organic acids are shown in Figure 4. The loading plots showed that lactic acid was distributed in the positive direction on the PC1 (accounting for 41% of the total variance), and vice versa, while the others were distributed in the negative direction (Figure 4B). These implied that lactic acid content in cheeses was negatively correlated with other organic acids in cheeses. However, because the score plots were not separated depending on the cheese variety, the organic acid content did not reflect the characteristics of the cheese variety (Figure 4A).

**FIGURE 5:**
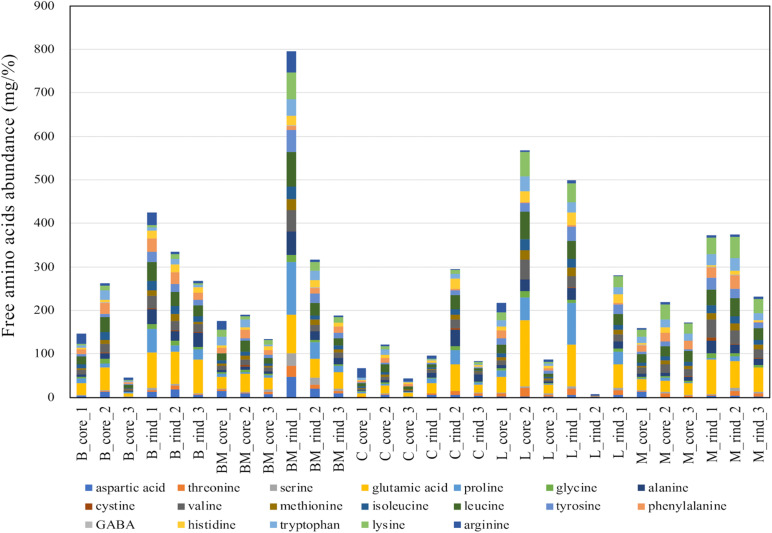
Abundances of free amino acids in 30 cheese samples. The total amount of free amino acids in each sample are shown in the barplot. B, Brie de Meaux; BM, Brie de Melun; C, Coulommiers; L, Langres; M, Maroilles.

**FIGURE 6:**
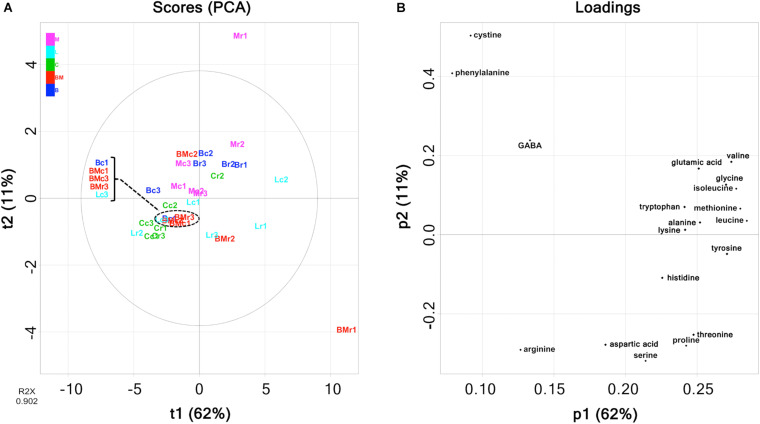
Principal component analysis (PCA) on the abundance of free amino acids in 30 cheese samples. **(A)** Score plot. **(B)** Loading plot. Bc, Brie de Meaux core; Br, Brie de Meaux rind; BMc, Brie de Melun core; BMr, Brie de Melun rind; Cc, Coulommiers core; Cr, Coulommiers rind; Lc, Langres core; Lr, Langres rind; Mc, Maroilles core; Mr, Maroilles rind.

Free amino acids were quantified using HPLC (Supplementary Table 2), and the amounts are summarized in Figure 5. Comparing each cheese variety, the amounts of amino acids in rind seemed to be higher than those in the core. However, it was difficult to infer the cheese characteristics because the amount of each free amino acid was variable for each cheese sample. As with organic acids, PCA was performed in an attempted to reveal the characteristics of free amino acid contents in each cheese variety. The PCA score and loading plots of the first two principal components of the free amino acids are shown in Figure 6. In the loading plots, all free amino acids were distributed in the positive direction on the PC1 (accounting for 62% of the total variance) (Figure 6B). This result implied that the total content of free amino acids was indicated by the PC1 and varied widely in each cheese sample. However, similar to organic acids, the score plots were scattered regardless of cheese type (Figure 6A). Thus, the free amino acid content in each cheese did not reflect the characteristics of the cheese variety.

**FIGURE 7:**
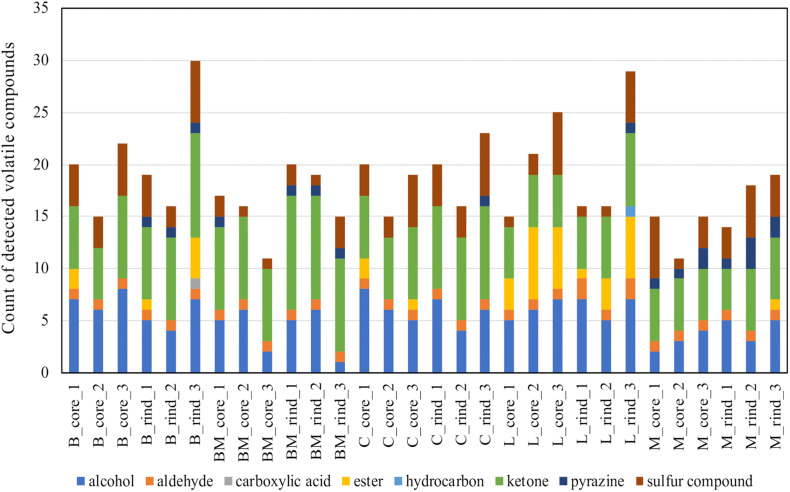
Count data of the detected volatile compounds in 30 cheese samples. Detected compounds were classified into eight categories and aggregated. Total counts of detected volatile compounds in each sample are shown in the barplot. B, Brie de Meaux; BM, Brie de Melun; C, Coulommiers; L, Langres; M, Maroilles.

**FIGURE 8:**
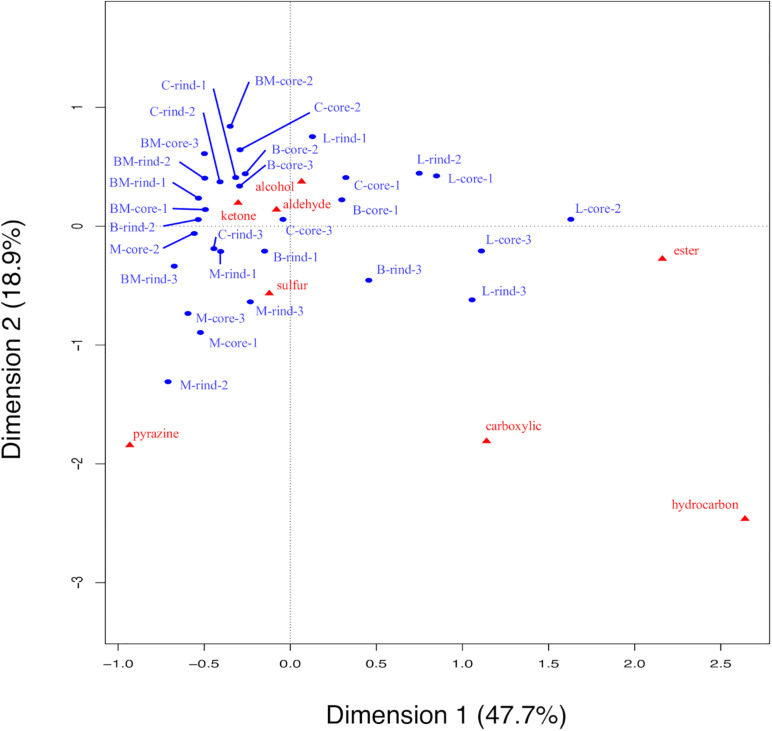
Biplot of principal coordinates and standard coordinates obtained by correspondence analysis (CA) for volatile compounds in 30 cheese samples. Detected compounds were classified into eight categories (alcohols, aldehydes, carboxylic acids, esters, hydrocarbons, ketones, pyrazines, and sulfur compounds). Blue plot indicates principal coordinates and red plot indicates standard coordinates. B, Brie de Meaux; BM, Brie de Melun; C, Coulommiers; L, Langres; M, Maroilles.

To detect volatile compounds in ripened cheese, HS-GC/MS was performed. By comparing the mass spectra and retention time, 58 volatile compounds were detected (Supplementary Tables 3, 4). These detected compounds were classified into eight categories (alcohols, aldehydes, carboxylic acids, esters, hydrocarbons, ketones, pyrazines, and sulfur compounds; Supplementary Table 5), and these categorical data were counted and are summarized in Figure 7. Total counts of detected volatile compounds in each cheese sample were generally approximately 20. Further, these categorical data were used for CA. The principal coordinate and the standard coordinate plot in the first dimension (explained 47.7% of the variance) and the second dimension (explained 18.9% of the variance) are shown in Figure 8. Surface mold-ripened cheese were clustered in the positive direction on the second dimension and tended to contain various ketones and alcohols. In bacterial smear-ripened cheese, Langres and Maroilles were found in the positive and negative directions on the first and second dimensions, respectively, and associated with ester and pyrazine. These results imply that Langres and Maroilles contain various types of esters and pyrazines as characteristic components, respectively.

Therefore, the characteristics of each cheese type is determined by the presence or absence of volatile compounds, and not by differences in the organic acid and free amino acid contents. Since this study used cheeses sold on the market, it was considered that the amounts of organic acids and free amino acids caused variation due to the difference in production lots and period until purchase, even though they had the same variety of cheeses. In contrast, qualitative analysis by classifying volatile compounds into eight categories revealed the tendency for cheese types, indicating the difference in the types of volatile compounds.

### Correlations Between Appearance of Bacteria and Constituent Metabolites in Ripened Cheeses

Thus far, although microbiota and volatile compounds suggested differences in cheese types, it was insufficient to determine the difference in cheese types by comparing organic acids and free amino acids only. Therefore, whether the characteristics of cheeses can be revealed by analyzing the relationship between the microbiota and metabolites in the cheese samples was examined.

**FIGURE 9:**
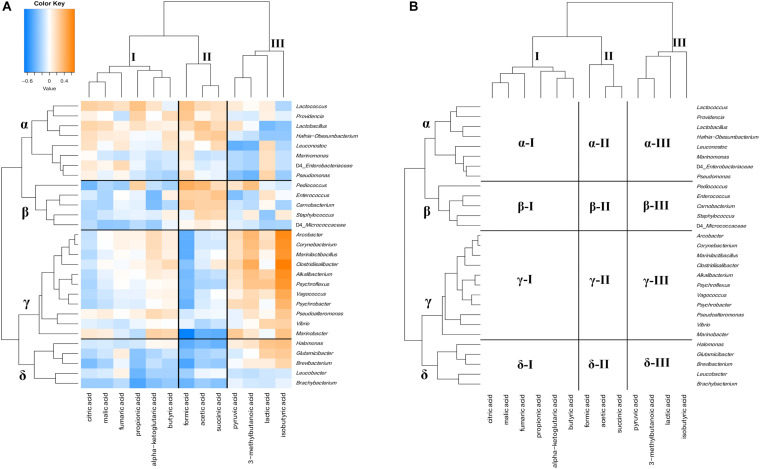
Hierarchical clustered map showing the Spearman correlation between bacteria genus and organic acids detected on 30 cheese samples. **(A)** Spearman correlation heat map. Orange indicates positive correlation and blue indicates negative correlation. **(B)** Hierarchically clustered map. Organic acids clusters are represented by I, II, and III. Bacteria clusters are represented by α, β, γ, and δ.

**FIGURE 10:**
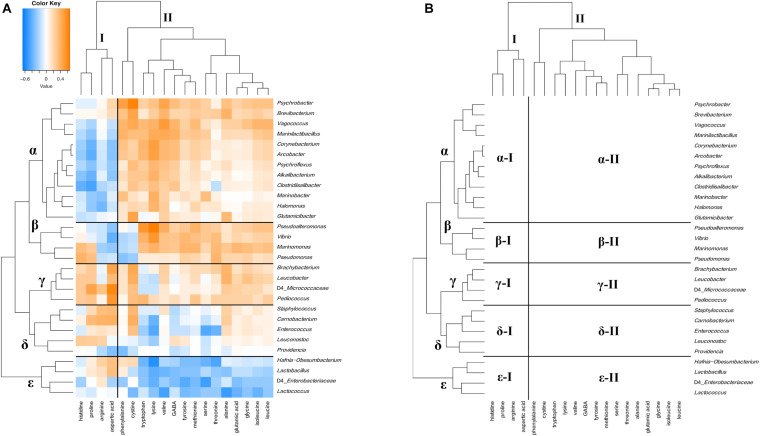
Hierarchical clustered map showing the Spearman correlation between bacteria genus and free amino acids detected on 30 cheese samples. **(A)** Spearman correlation heat map. Orange indicates positive correlation and blue indicates negative correlation. **(B)** Hierarchically clustered map. Free amino acids clusters are represented by I, II, and III. Bacteria clusters were represented by α, β, γ, δ, and ε.

**FIGURE 11:**
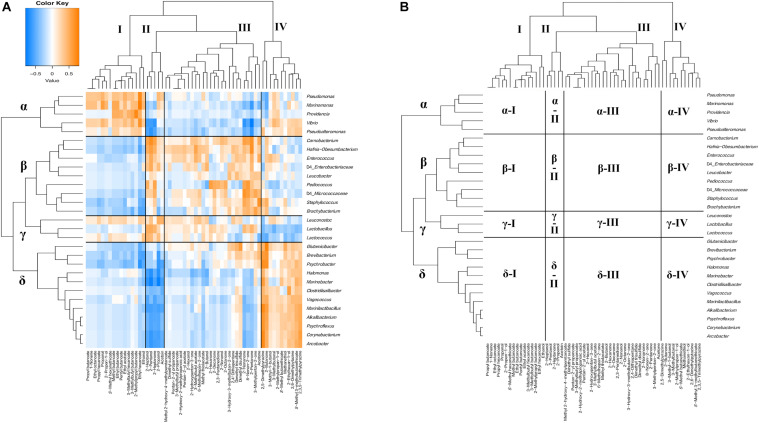
Hierarchical clustered map showing the Spearman correlation between bacteria genus and volatile compounds detected on 30 cheese samples. **(A)** Spearman correlation heat map. Orange indicates positive correlation and blue indicates negative correlation. **(B)** Hierarchically clustered map. Volatile compounds clusters are represented by I, II, III, and IV. Bacteria clusters are represented by α, β, γ, and δ.

The relationship between bacterial communities and metabolites in ripened cheeses was verified by hierarchical cluster analysis based on Spearman’s correlation coefficient. As a result, the correlation tendency of the metabolites differed depending on the frequency of appearance of each bacterium. The degree of correlation between the appearance of bacteria and organic acids, free amino acids, and volatile compounds is shown in Figures 9–11, respectively.

Hierarchical cluster analysis divided organic acids and bacteria into three clusters (I, II, and III) and four clusters (α, β, γ, and δ), respectively (Figures 9A,B). Cluster I contained six types of organic acids, such as citric acid and propionic acid. Cluster II contained formic acid, acetic acid, and succinic acid, and cluster III grouped pyruvic acid, 3-methylbutanoic acid, lactic acid, and isobutyric acid. In bacterial clusters, cluster α was mainly composed of LAB, such as *Lactococcus*. Cluster β mainly grouped NSLAB, such as *Pediococcus*. Cluster γ was mainly composed of MB, HALAB, and *Corynebacterium* of the phylum *Actinobacteria*. Cluster δ was mainly composed of *Actinobacteria*, except for *Corynebacterium*. Cluster α bacteria were negatively correlated with some components constituting cluster III (α-III). Cluster β bacteria, especially *Pediococcus*, positively correlated with the cluster II component (β-II: *Pediococcus* - acetic acid and formic acid) and negatively correlated with the cluster I component (β-I: *Pediococcus* - citric acid). Cluster γ bacteria such as HALAB and *Psychrobacter* were positively correlated with the cluster III component, especially in isobutyric acid (γ-III), and negatively correlated with the cluster II component, especially in formic acid (γ-II). Cluster δ bacteria tended to show negative correlations with cluster I and II components (δ-I and δ-II). Significant correlations between the appearance of bacteria and organic acids are shown in Supplementary Tables 6, 7.

Free amino acids and bacteria were divided into two clusters (I and II) and five clusters (α, β, γ, δ, and ε), respectively (Figures 10A,B). Cluster I contained four free amino acids, such as aspartic acid, and cluster II contained the other free amino acids. In bacterial clusters, cluster α was composed of *Psychrobacter*, *Halomonas*, and *Marinobacter*, which belong to MB, *Marinilactibacillus*, *Alkalibacterium*, and *Vagococcus*, which belong to HALAB and *Brevibacterium*, *Glutamicibacter*, and *Corynebacterium* of the phylum *Actinobacteria*. Cluster β was composed of *Pseudoalteromonas*, *Marinomonas*, *Vibrio*, and *Pseudomonas*, which belong to MB. Cluster γ was composed of *Brachybacterium* and *Leucobacter* of the phylum *Actinobacteria* and *Pediococcus* is known as NSLAB. Clusters δ and ε were mainly composed of NSLAB, such as *Enterococcus* and *Carnobacterium*, and LAB such as *Lactococcus*, respectively. Cluster α bacteria negatively correlated with the cluster I component (α-I: *Alkalibacterium* – proline), but positively correlated with the cluster II component (α-II: *Brevibacterium –* cystine, *Psychrobacter* – phenylalanine, and *Halomonas* – lysine). Cluster β bacteria positively correlated with cluster II components, except for cystine and phenylalanine (β-II: *Pseudoalteromonas* - methionine and *Vibrio* – lysine). Clusters γ and δ positively correlated with the cluster I component, especially aspartic acid (γ-I: *Pediococcus* - aspartic acid and δ-I: *Carnobacterium* - aspartic acid). In addition, these clusters were positively correlated with cystine (γ-II: *Brachybacterium* – cystine and δ-II: *Carnobacterium* – cystine). Cluster ε bacteria was negatively correlated with the cluster II component (ε-II). Significant correlations between the appearance of bacteria and free amino acids are shown in Supplementary Tables 8, 9.

Volatile compounds and bacteria were divided into four clusters based on their correlations (I, II, III, and IV and α, β, γ, and δ) (Figures 11A,B). Cluster I contained various kinds of esters such as ethyl acetate and ethyl hexanoate. Cluster II contained 2-pentanol, 2-heptanol, 2-propanol, 2-heptanone, and acetoin. Cluster III contained various kinds of alcohols and ketones, such as 2-nonanol and acetone, in addition to sulfur components such as dimethyl disulfide. Cluster IV mainly contained 2-butanone, pyrazines such as 2,5-dimethylpyrazine, and sulfur compounds, such as methanethiol. In bacterial clusters, cluster α was composed of MB, such as *Marinomonas* and *Pseudoalteromonas*. Cluster β was composed of *Carnobacterium*, *Enterococcus*, and *Pediococcus*, which are known as NSLAB; and *Brachybacterium* and *Leucobacter* of the phylum *Actinobacteria*. Cluster γ was composed of *Lactococcus*, *Lactobacillus*, and *Leuconostoc*, which are known as LAB. Cluster δ was composed of *Psychrobacter*, *Halomonas*, and *Marinobacter*, which belong to MB; *Marinilactibacillus*, *Alkalibacterium*, and *Vagococcus*, which belong to HALAB; and *Brevibacterium*, *Glutamicibacter*, and *Corynebacterium*, of the phylum *Actinobacteria*. Cluster α bacteria positively correlated with the cluster I component (α-I: *Marinomonas* – ethyl acetate) and negatively correlated with cluster II and cluster III components (α-II: *Pseudoalteromonas* – 2-pentanol and α-III: *Marinomonas* – acetone). In contrast, cluster β bacteria negatively correlated with the cluster I component (β-I: *Carnobacterium* – ethyl hexanoate) and positively correlated with cluster II and cluster III components (β-II: *Pediococcus* – 2-heptanol and β-III: *Brachybacterium* - acetone). Cluster γ bacteria were positively correlated with cluster II and cluster III components (γ-II: *Lactococcus* – 2-pentanol and γ-III: *Lactobacillus* – 2-pentanone). Moreover, cluster γ bacteria were negatively correlated with the cluster IV component (γ-IV: *Lactococcus* - 2,5-dimethylpyrazine). Cluster δ bacteria negatively correlated with cluster I, cluster II, and cluster III components (δ-I: *Glutamicibacter* – ethanol, δ-II: *Marinilactibacillus* – 2-pentanol, and δ-III: *Halomonas* – 2-butanol) and positively correlated with the cluster IV component (δ-IV: *Glutamicibacter* - methanethiol and *Marinilactibacillus* − 2,5-dimethylpyrazine). Among them, *Glutamicibacter* and *Brevibacterium* tended to show positive correlations with dimethyl disulfide, dimethyl trisulfide, and acetone in the cluster III component (δ-III). Significant correlations between the appearance of bacteria and volatile compounds are shown in Supplementary Tables 10, 11.

As mentioned above, PCA based on the organic acid and free amino acid contents in each cheese did not reflect the characteristics of the cheese variety. However, Spearman correlation analysis revealed that the variation in cheese containing metabolites was associated with the bacterial abundance and genus differences. Furthermore, hierarchical clustering based on Spearman correlation showed that the volatile compounds characterized by CA were also strongly associated with the bacterial abundance and differences in genera. In particular, the difference between LAB and MB was remarkable with regard to all of the tested metabolites. Thus, the presence of LAB and MB was considered to be crucial for ripened cheese components.

### Correlations Between the Appearance of Fungi and Constituent Metabolites in Ripened Cheeses

Similar to bacteria, the relationship between fungal communities and the metabolites in the ripened cheeses was verified using hierarchical cluster analysis based on Spearman’s correlation coefficient (Supplementary Figures 2–4). The correlations between organic acids and fungi were separated into different clusters by hierarchical analysis, although the effective size was weak overall (Supplementary Figure 2). Organic acids were divided into three clusters (I, II, and III). Cluster I contained isobutyric acid and lactic acid. Cluster II contained 10 kinds of organic acids, such as 3-methylbutanoic acid. Cluster III was assigned to formic acid. Fungi were divided into four clusters (α, β, γ, and δ). Cluster α was composed of *Scopulariopsis* and *Cyberlindnera*. Cluster β was composed of *Debaryomyces*. Cluster γ was composed of *Penicillium*, *Kluyveromyces*, and other two genera. Cluster δ was composed of *Dipodascus* and *Saturnispora*. Cluster α fungi were negatively correlated with the cluster I components (α-I). *Debaryomyces* was positively correlated with isobutyric acid (β-I) and negatively correlated with formic acid (β-III). In contrast, cluster γ and cluster δ fungi, such as *Penicillium* and *Dipodascus*, were negatively correlated with isobutyric acid (γ-I and δ-I) and positively correlated with formic acid (γ-III and δ-III). In addition, cluster δ fungi were negatively correlated with the cluster II component (δ-II). Significant correlations between the appearance of fungi and organic acids are shown in Supplementary Tables 12, 13.

The correlation between free amino acids and fungi was mostly negative (Supplementary Figure 3). The free amino acids were divided into two clusters (I and II). Cluster I contained 13 free amino acids, such as lysine, serine, and threonine. Cluster II contained other free amino acids, such as aspartic acid and proline. Fungi were divided into three clusters (α, β, and γ). Cluster α was assigned to *Debaryomyces*. Cluster β was composed of *Penicillium*, *Dipodascus*, and other two genera. Cluster γ was composed of *Kluyveromyces*, *Saturnispora*, and other three genera. *Debaryomyces* was positively correlated with lysine in the cluster I component (α-I) and negatively correlated with the cluster II component (α-II). In cluster β fungi, *Penicillium* and *Scopulariopsis* were positively correlated with aspartic acid (β-II). Additionally, cluster β fungi, except *Scopulariopsis*, tended to show negative correlations with the cluster II component (β-II: *Penicillium* – tryptophan and *Pichia* – lysine). Cluster γ fungi were negatively correlated with the overall cluster I component (γ-I: *Candida* – serine and *Kluyveromyces* - lysine). Significant correlations between the appearance of fungi and free amino acids are shown in Supplementary Tables 14, 15.

In the hierarchical analysis of the correlation between volatile compounds and fungi, volatile compounds were divided into three clusters (I, II, and III) (Supplementary Figure 4). Cluster I contained various esters such as ethyl hexanoate and also contained 3-methylbutanal, dimethyl disulfide, and pyrazines. Cluster II contained 2-pentanol, 2-heptanol, 2-pentanone, and 2-heptanone. Cluster III contained ketones such as 2-nonanoe, alcohols such as 2-butanol, esters such as 3-methylbutyl acetate, and sulfur compounds such as methyl thioacetate. Fungi were divided into three clusters (α, β, and γ). Cluster α was composed of *Dipodascus*, *Kluyveromyces*, and other four genera. Cluster β was composed of *Penicillium* and *Scopulariopsis*. Cluster γ was composed of *Debaryomyces*. Cluster α and cluster β fungi showed strong positive correlations with the cluster II component (α-II: *Candida* – 2-pentanol and β-II: *Penicillium* – 2-pentanone). Cluster α fungi were also positively correlated with some compounds in cluster III (α-III: *Dipodascus* – acetoin). *Penicillium* and *Scopulariopsis* were positively correlated with ketones in cluster III (β-III: *Penicillium* – 2-nonanone and *Scopulariopsis* – 8-nonen-2-one). Moreover, cluster α and cluster β fungi negatively correlated with cluster I components (α-I: *Dipodascus* – 2,5-dimethylpyrazine, and β-I: *Penicillium* – ethyl acetate). *Debaryomyces* was negatively correlated with cluster II and cluster III components (γ-II: *Debaryomyces* – 2-pentanol and γ-III: *Debaryomyces* – 2-nonanone) and positively correlated with 2,5-dimethylpyrazine and 2,3,5-trimethylpyrazine (γ-I). Significant correlations between the appearance of fungi and volatile compounds are shown in Supplementary Tables 16, 17.

These results showed that the fungi were particularly correlated with secondary alcohols such as 2-pentanol and 2-heptanol and methyl ketones such as 2-pentanone and 2-heptanone. Hierarchical cluster analysis based on the correlation between fungi and metabolites in ripened cheeses often showed that clusters were assigned to a single fungal genus. This may be a reflection of the fact that only a few fungi were found to dominate the ripened cheese. However, the number and strength of correlation suggest that the fungi strongly affected to the accumulation of limited kinds of components in ripened cheeses.

## Discussion

This study was conducted to verify which microorganisms and/or metabolites are affected by the difference in ripening method and to clarify a correlation between the microbiota and metabolites of surface mold-ripened cheese and bacterial smear-ripened cheese. The NaCl concentration (1.0–2.0%) and pH (5.0–7.4) in the core of cheeses tested could stimulate the growth of non-halophilic, halophilic, acidophilic, and/or neutrophilic microorganisms. On the other hand, the NaCl concentration (2.0–3.0) and pH (7.5–8.3) in the rind of cheeses could stimulate the growth of halotolerant, halophilic, neutrophilic, and/or alkaliphilic microorganisms, except for the pH of Langres (pH 6.7–6.8). Considering core and rind together, cheese samples can be regarded as a suitable environment in which various microorganisms grow, in terms of salinity and pH. Thus, although the salinity and pH of the cheese samples were generally similar, metagenomic analysis showed the specific microbiota that reflect different types of cheese. Surface mold-ripened cheese and bacterial smear-ripened cheeses were characterized by LAB and *Penicillium* and MB, HALAB, and *Debaryomyces*, respectively. However, the profile of metabolites in each cheese did not reflect the difference in cheese type, except for the qualitative analysis of volatile compounds. In contrast, Spearman correlation analysis revealed the relationships between microbiota and metabolites in ripened cheese. This result obtained by combining metagenomics and metabolomics shows the superiority of the integrated approach.

In general, fungi are known to play an important role in the deacidification and the development of flavor in ripened cheese (McSweeney, 2004; Gori et al., 2007; Monnet et al., 2015; Fröhlich-Wyder et al., 2019). In this study, *Penicillium*, *Kluyveromyces*, and *Candida*, which dominated surface mold-ripened cheese, showed positive correlations with secondary alcohols and ketones, especially 2-pentanol, 2-heptanol, 2-pentanone, and 2-heptanone, while *Debaryomyces*, which dominated smear-ripened cheese, showed a negative correlation. These results reflected the differences in cheese type derived from the fungal community. Previous studies have shown that secondary alcohols and methyl ketones were detected in surface mold-ripened cheese (Sablé and Cottenceau, 1999; Spinnler, 2017), and *P. camemberti* and *G. candidum* may produce methyl ketones (Thierry et al., 2017). In addition, *Penicillium* spp. possess the pathway that alkan-2-ones may be reduced to the corresponding secondary alcohol by fatty acid metabolism (McSweeney and Sousa, 2000). These findings are consistent with those of our study. Therefore, the correlation analysis between fungi and metabolites in ripened cheeses was able to show the relationship between fungi and secondary alcohols and methyl ketones.

Further, hierarchical cluster analysis of the correlations between bacterial communities and metabolites in ripened cheeses revealed that the appearance of specific bacteria was associated with the presence of organic acids, free amino acids, and volatile compounds. Our study showed that LAB groups, such as *Lactococcus*, were positively correlated with alcohols and ketones. Moreover, *Pediococcus*, *Enterococcus*, and *Carnobacterium*, which are known as NSLAB, also correlated with formic acid, acetic acid, and aspartic acid. Among the components that showed positive correlation with LAB in our study, ethanol, 2-pentanol, 2-nonanol, 3-methyl-1-butanol, acetone, 2-pentanone, 2-heptanone, 2-octanone, 2-nonanone, and acetoin are known to contribute to the flavors of cheeses such as mold-ripened cheese and surface-ripened cheese (Sablé and Cottenceau, 1999). Additionally, acetate, formate, and ethanol are generated from lactate by NSLAB, especially pediococci (McSweeney, 2004). A previous study investigating the influence of *Pediococcus acidilactici*, which was isolated from hard-type Swiss cheese, in model cheeses showed that higher acetate, 2-butanone, and 2-butanol levels were present in cheese with *P. acidilactici* than in the control cheese (Eugster et al., 2019). In the research focused on SLAB, Ruggirello et al. (2018) revealed that early ripened semi-hard Toma-like miniature cheese samples, that were produced using commercial starter cultures including *Lactococcus lactis* subsp. *lactis*, were characterized by high concentration of ketones such as 1-hydroxy-2-propanone, acetoin, acetone, and diacetyl; and alcohols such as 2-ethylhexan-1-ol, 2,3-butanediol, 3-methyl-2-buten-1-ol, and ethanol. This finding showed a similar relevance to our study in that LAB correlate with ketones and alcohols, although it is not a perfect match for the types of volatile compounds. Moreover, Sgarbi et al. (2013) evaluated the ability of two *Lactobacillus casei* and two *Lactobacillus rhamnosus* strains isolated from hard-type Parmigiano Reggiano cheese to produce volatile flavor compounds on cheese-based medium (CBM) and on starter LAB lysed cell medium (LCM), and found that the volatile compounds after bacterial growth on CBM were characterized by the presence of compounds such as acetoin, diacetyl, acetone, and other ketones, as well as benzaldehyde and acetic acid. In our study, diacetyl and benzaldehyde were not detected; however, the association between LAB and ketones and acetic acid agreed with the findings of previous studies, regardless of the type of cheese or the origin of bacterial isolates.

*Actinobacteria* are commonly found in ripened cheeses, especially *Brevibacterium linens*, which are important microorganisms in smear-ripened cheese (Rattray and Fox, 1999; Bertuzzi et al., 2018). Furthermore, *Corynebacterium*, *Glutamicibacter* (*Arthrobacter*), *Brachybacterium*, and *Leucobacter* were also detected in various cheeses (Irlinger et al., 2015). Among the five varieties of ripened cheese in this study, Langres hardly harbored *Actinobacteria*. These bacteria, such as *Brevibacterium* sp. and *Corynebacterium* sp., are acid-sensitive and begin to grow when the pH is approximately 5.5–6.0 in cheese (Monnet et al., 2015). The pH of Langres was 5.0–5.2 and 6.7–6.8 in the core and rind, respectively, lower than that in the other samples. Therefore, this might be one of the reasons for the lower proportion of *Actinobacteria* in Langres.

In previous studies, *B. linens* and *Micrococcaceae* (to which *Glutamicibacter* belongs) found in red smear cheese are considered to be the main producers of sulfur compounds (Rattray and Fox, 1999; Bertuzzi et al., 2018). It has been reported that *B. linens* produces fatty acids, alcohols, methyl ketones, pyrazines, sulfur compounds, and cyclic compounds in culture media (Rattray and Fox, 1999). In addition, *Brevibacterium* spp. isolated from hard-type Beaufort cheese produced *S*-methyl thioesters using short-chain fatty acids or branched-chain amino acids as precursors (Rattray and Fox, 1999; Sourabié et al., 2012). Deetae et al. (2007) validated that the *Brachybacterium* strain isolated from surface-ripened cheese produced high amounts of ketones on casamino acid medium.

*Actinobacteria* were mostly divided into two tendencies based on correlation analysis with metabolites in our study. *Brevibacterium*, *Glutamicibacter*, and *Corynebacterium* were similar to MB and HALAB, while *Brachybacterium* and *Leucobacter* were similarly correlated with LAB. The former showed a positive correlation with sulfur compounds such as methanethiol, dimethyl disulfide, dimethyl trisulfide, *S*-methyl butanethioate, and *S*-methyl 3-methylbutanethioate and pyrazines, which agrees with previous findings. The latter showed a positive correlation with aspartic acid, cystine, alanine, and ketones. The correlation of *Brachybacterium* is consistent with the study of Deetae et al. (2007), indicating that it may also contribute to cheese flavor along with LAB regardless of the type of cheese or the origin of bacterial isolates.

Marine bacteria have been reported to be widespread in cheese microbial communities using high-throughput sequencing technologies (Irlinger et al., 2015). These microorganisms, which are known to be halophilic and psychrotolerant, have previously been detected in brine as well as marine environments (Holmström and Kjelleberg, 1999; Reen et al., 2006; Marino et al., 2017; Haastrup et al., 2018; Vermote et al., 2018). Considering from these findings, it is reasonable to assume that MB are introduced from the sea salts used for the brine and are adapted to the cheese-making environment through the salting step (Irlinger et al., 2015). All of the samples in this study had a slightly saline environment (1.0–3.0%) in which MB can grow. Moreover, our study confirmed the dominance of MB in cheese samples, especially in bacterial smear-ripened cheeses. This indicates that washing with brine in the ripening process is an important factor to encourage MB to dominate cheese microbiota. However, Langres and Maroilles used in our study were dominated by different MB in spite of being the same type of cheese. As shown in this study, cheese is occupied with adventitious microorganisms that are considered to be transferred from milk and cheese-making environments as well as deliberately added microorganisms (Montel et al., 2014; Irlinger et al., 2015; Gobbetti et al., 2018). Interestingly, a study that investigated the microbial ecosystems of two artisan cheesemaking facilities that produce a similar range of products consisting of fresh, bloomy-rind, and smear-ripened cheese, has shown that cheese and aging-rooms in facility A were dominated by *Brevibacterium*, *Staphylococcus*, *Psychrobacter*, and *Corynebacterium*, whereas those in facility B were dominated by *Pseudoalteromonas*, *Vibrio*, and *Vibrionaceae* (Bokulich and Mills, 2013). This evidence implies that the difference in microbiota between Langres and Maroilles in this study reflects the effects of environmental microbiota.

Although studies on MB in cheeses are limited, some findings have recently demonstrated its contribution to cheese flavor. *Psychrobacter* sp., isolated from surface-ripened French cheese, produced branched aldehydes, alcohols, and esters on casamino acid medium (Deetae et al., 2007) and smear soft cheese experimentally inoculated with *Psychrobacter celer*, which increases throughout the ripening, showed higher concentrations of aldehydes, ketones, and sulfur compounds (Irlinger et al., 2012). *Pseudoalteromonas* is known to possess cold-adapted enzymes, which may contribute to the development of cheese flavor during ripening, storage, and transportation at low temperatures (De Pascale et al., 2008, 2010; Wolfe et al., 2014). Furthermore, Wolfe et al. (2014) reported the *mgl* sequences (coding methionine-gamma-lyase [EC:4.4.1.11]) with high sequence similarity to various *gamma-Proteobacteria* in naturally aged cheeses from both Europe and North America by shotgun metagenomics.

In our study, MB were strongly associated with cheese metabolites, especially volatile compounds. *Pseudoalteromonas*, *Vibrio*, and *Marinomonas* were positively correlated with esters, while *Psychrobacter*, *Marinobacter*, and *Halomonas* were positively correlated with pyrazines along with HALAB. Previous studies have shown that esters are synthesized by the reaction of free fatty acids and alcohols, and this reaction is considered to be caused by LAB, *G. candidum*, and *Pseudomonas fragi* (Bertuzzi et al., 2018; Khattab et al., 2019). Our study indicated that MB, such as *Pseudoalteromonas*, *Vibrio*, and *Marinomonas*, were also associated with esters as well as *Pseudomonas*. Although pyrazines have been found in ripened cheeses, biochemical mechanisms are poorly understood (McSweeney and Sousa, 2000). Therefore, the existence of MB and HALAB is considered very important for the elucidation of the pyrazine production mechanism.

As with MB, the existence of HALAB in smear-ripened cheeses has been reported previously (Mounier et al., 2017). Previous experiments have shown that *Marinilactibacillus psychrotolerans* B-7-9-5 produces acetate from lactate during ripening in Brie-type model cheeses, and *Vagococcus lutrae* 9A8 produces acetate from lactose under aerobic conditions (Unno et al., 2020; Suzuki et al., 2021). Nevertheless, HALAB were less associated with acetic acid and showed a positive correlation with isobutyric acid in this study. In this way, while the correlations between bacteria and metabolites in our study were broadly in agreement with previous studies, some correlations were not. The flavor and texture characteristics of cheeses occur through microbiological and biochemical events, such as lipolysis, proteolysis, and metabolism of residual lactose, lactate, and citrate during ripening (McSweeney and Sousa, 2000; McSweeney, 2004; Khattab et al., 2019). Moreover, cheese can be regarded as a biocomplex ecosystem colonized by a diverse group of microorganisms that interact with each other (Irlinger and Mounier, 2009; Irlinger et al., 2015; Khattab et al., 2019). These effects also occurred in our study, and it is considered that some correlations showed discrepancies with previous studies, which were verified with a single strain.

Considering the findings in this study along with the previous findings, hierarchical cluster analysis based on the correlation clarified that the presence or absence and abundance of specific bacteria such as LAB, MB, HALAB, and *Actinobacteria* are related to the formation of specific cheese components. This indicates that the relationship between the existing bacteria and components of cheeses is generally similar, even if the type of cheese and the origin of the bacteria are different. Furthermore, even though this study used cheeses after ripening sold in food markets and did not monitor the ripening process of microorganisms and metabolites, the specific relationships between microorganisms and metabolites could be clearly shown. Thus, the correlation analysis method applied in this study can be considered to be appropriate for linking the microbiota and components of various cheeses. Based on this perspective, the role of MB in cheese has not been revealed previously; however, this study suggested that MB may play an important role in inducing cheese characteristics in flavor formation. Although there are many non-starter microorganisms for which their features have not been revealed in cheese, it can be regarded that accumulating data showing the relationship between microorganisms and metabolites in various cheese types will improve our systematic understanding of cheese consortia including adventitious microbiota. In this study, our results showed correlations between microorganisms and metabolites in ripened cheeses, but not causal relations. In addition, the mechanism of the competitive and symbiotic effects among the microorganisms appeared in ripened cheeses remains unclear. Thus, further studies, such as demonstration experiments using cheese isolate strains or metatranscriptomic analysis in the cheese ripening process, are needed to confirm the relationships shown in this study. However, methodologies and results obtained in this study will provide the basis for elucidating cheese consortia widely and help in the selection of cheese adjunct cultures for the accumulation of specific flavors in the future.

## Data Availability Statement

The datasets generated for this study can be found in online repositories. The names of the repository/repositories and accession number(s) can be found below: https://www.ddbj.nig.ac.jp/, DRA011532.

## Author Contributions

RU and MI wrote the manuscript. RU performed the experiments. RU, TS, and MM contributed to the data analysis. MI coordinated the study. All authors read and approved the final manuscript.

## Conflict of Interest

The authors declare that the research was conducted in the absence of any commercial or financial relationships that could be construed as a potential conflict of interest.

## Footnotes

1 http://www.nist.gov
