# Supplementary material for: Evaluation of the Relationships Between Microbiota and Metabolites in Soft-Type Ripened Cheese Using an Integrated Omics Approach
Source: Front Microbiol. 2021 Jun 8;12:681185. doi: 10.3389/fmicb.2021.681185 (PMC8219077; doi:10.3389/fmicb.2021.681185)
Supplement: Supplementary file 1 [file Data_Sheet_1.pdf]

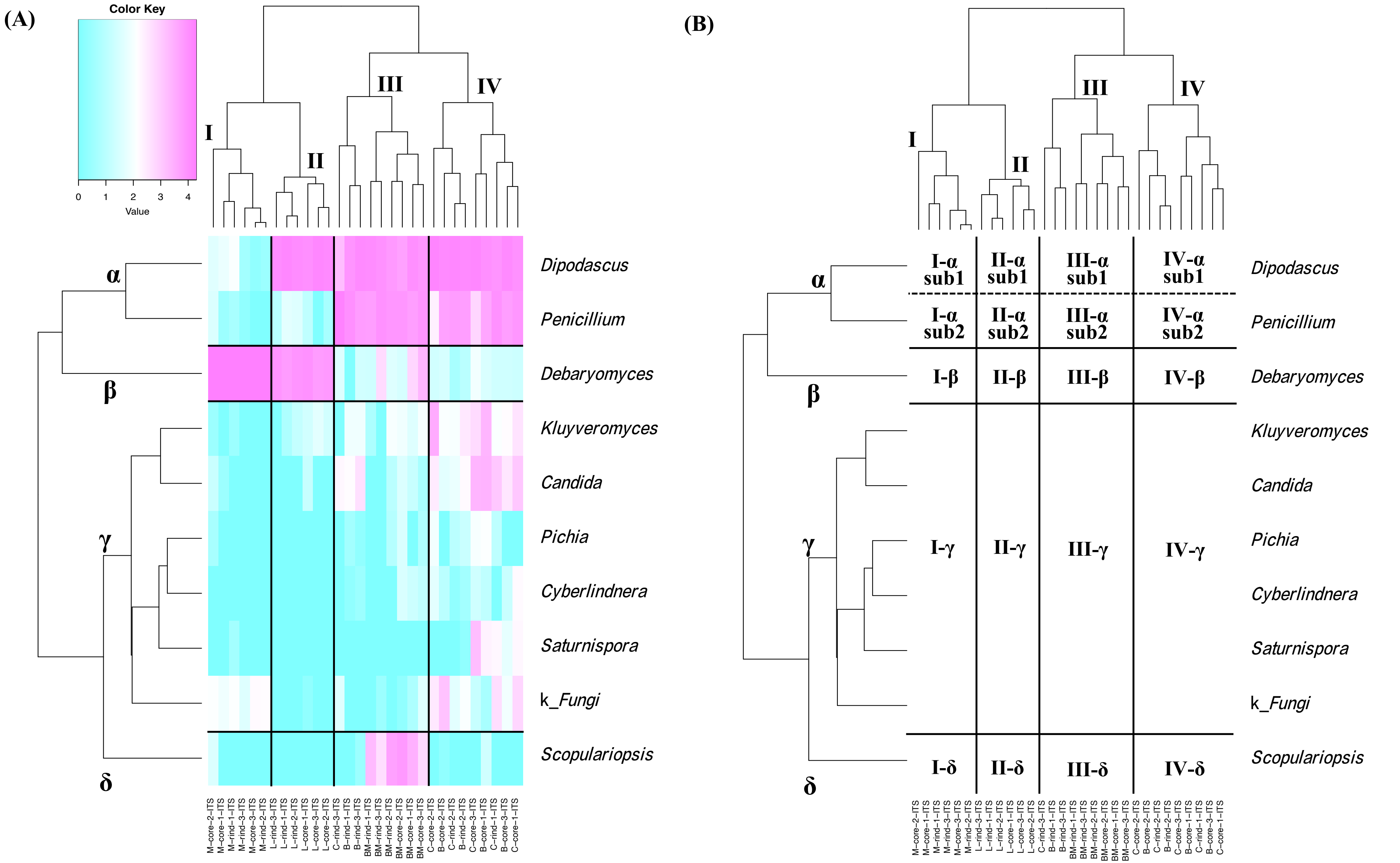

Fig. S1. Hierarchical clustered heat map based on the relative abundance of fungi genus detected on 30 cheese samples. **(A)** Relative abundance of fungi genus is shown on heat map with dendrogram. Only fungi with relative abundance > 0.5% in at least one sample are shown. Purple indicates high occupancy and light blue indicates low or non-existent occupancy. **(B)** Hierarchically clustered map. Cheese sample clusters are represented by I, II, III, and IV. Fungi clusters are represented by α, β, γ, and δ. Cluster α is further divided into sub1 and sub2. B: Brie de Meaux; BM: Brie de Melun; C: Coulommiers; L: Langres; M: Maroilles

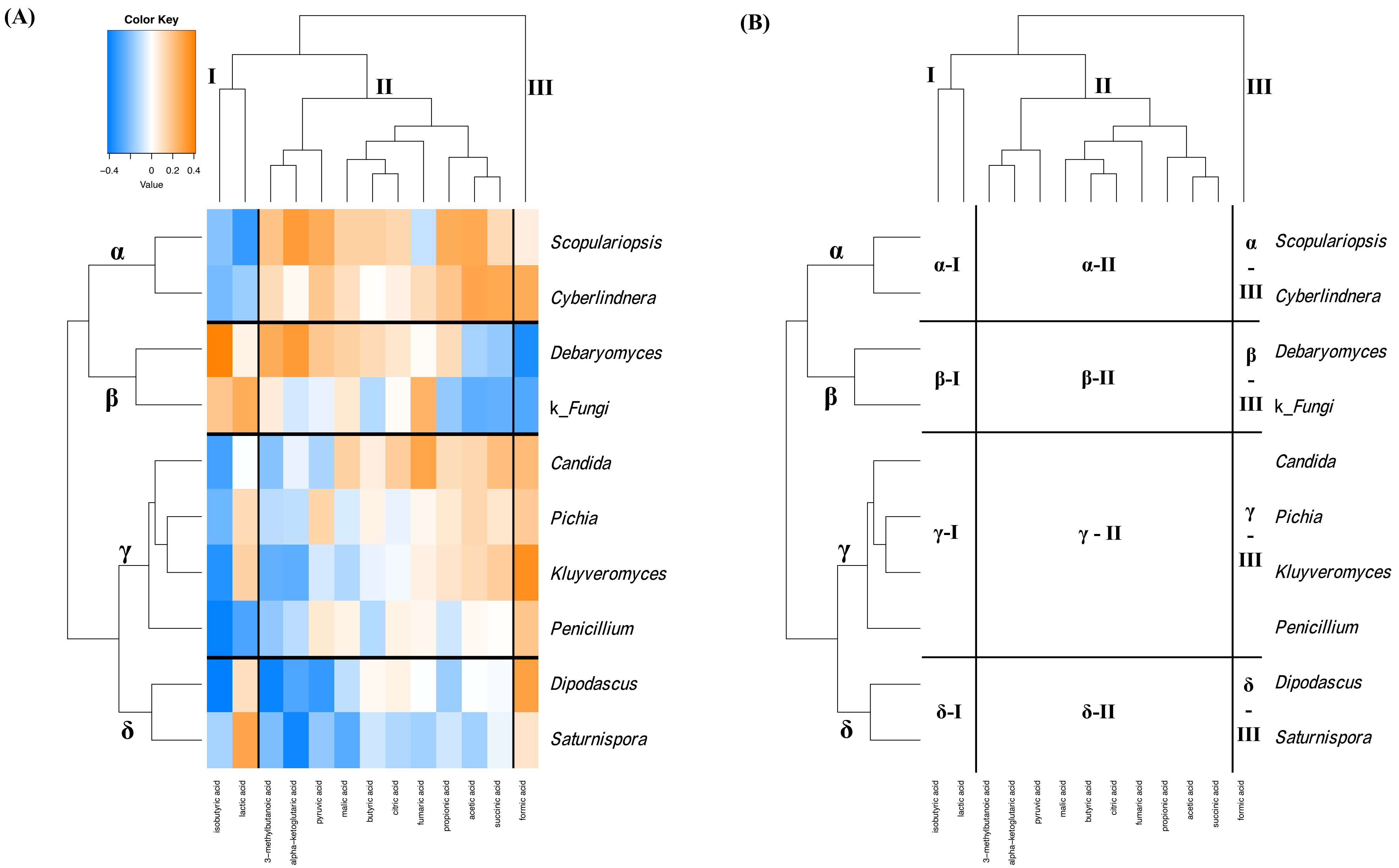

Fig. S2. Hierarchical clustered map showing the Spearman correlation between fungi genus and organic acids detected on 30 cheese samples. **(A)** Spearman correlation heat map. Orange indicates positive correlation and blue indicates negative correlation. **(B)** Hierarchically clustered map. Organic acids clusters are represented by I, II, and III. Fungi clusters are represented by α, β, γ, and δ.

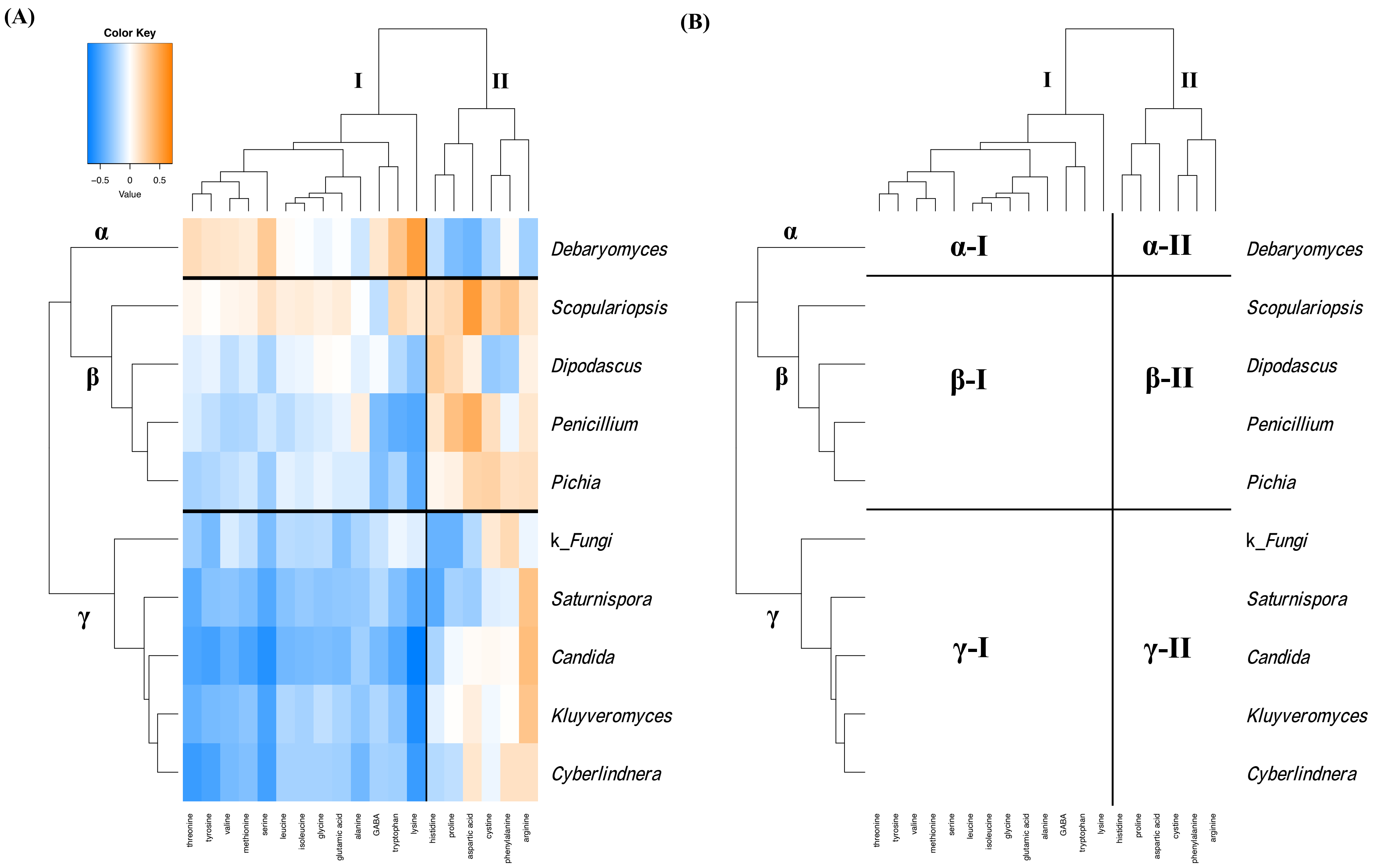

Fig. S3. Hierarchical clustered map showing the Spearman correlation between fungi genus and free amino acids detected on 30 cheese samples. **(A)** Spearman correlation heat map. Orange indicates positive correlation and blue indicates negative correlation. **(B)** Hierarchically clustered map. Free amino acids clusters are represented by I and II. Fungi clusters are represented by α, β, and γ.

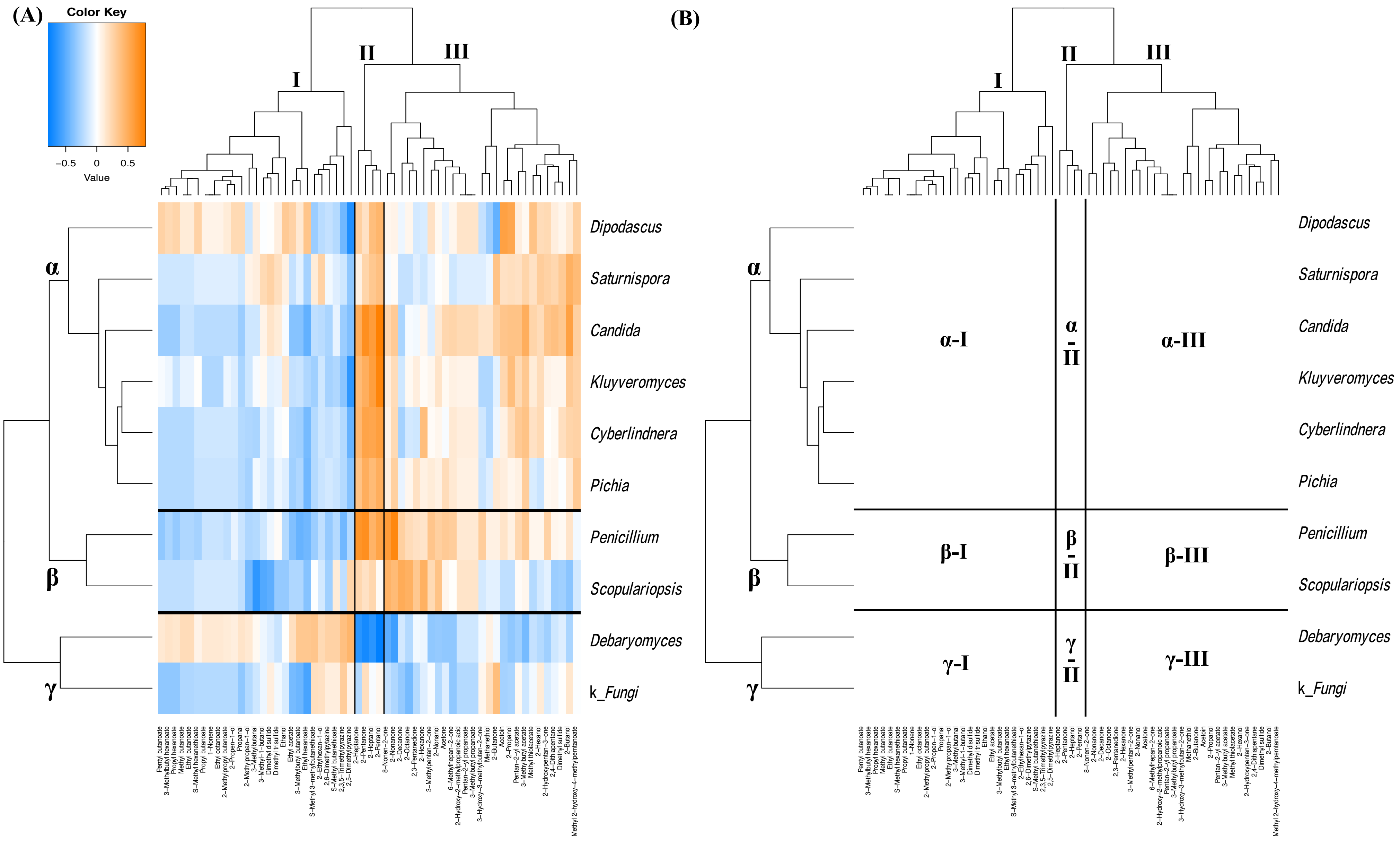

Fig. S4. Hierarchical clustered map showing the Spearman correlation between fungi genus and volatile compounds detected on 30 cheese samples. **(A)** Spearman correlation heat map. Orange indicates positive correlation and blue indicates negative correlation. **(B)** Hierarchically clustered map. Volatile compounds clusters are represented by I, II, and III. Fungi clusters are represented by α, β, and γ.
