# Supplementary material for: Evaluation of the Relationships Between Microbiota and Metabolites in Soft-Type Ripened Cheese Using an Integrated Omics Approach
Source: Front Microbiol. 2021 Jun 8;12:681185. doi: 10.3389/fmicb.2021.681185 (PMC8219077; doi:10.3389/fmicb.2021.681185)
Supplement: Supplementary file 2 [file Data_Sheet_2.PDF]

Table S1. Organic acids abundance, expressed as mg/%,  in 30 cheese samples. B: Brie de Meaux; BM: Brie de Melun; C: Coulommiers; L: Langres; M:Maroilles

| Sample | Alpha-Ketoglutaric acid | Citric acid | Pyruvic acid | Malic acid | Succinic acid | Lactic acid | Fumaric acid | Formic acid | Acetic acid | Propionic acid | Isobutyric acid | Butyric acid | 3-Methylbutanoic acid |
|---|---|---|---|---|---|---|---|---|---|---|---|---|---|
| B-core-1 | 145.6 | 65.1 | 0.0 | 12.3 | 215.3 | 14.9 | 0.0 | 11.1 | 504.1 | 18.6 | 0.0 | 29.5 | 0.0 |
| B-core-2 | 114.0 | 61.1 | 0.0 | 23.2 | 53.5 | 47.5 | 5.3 | 6.9 | 232.9 | 0.0 | 0.0 | 0.0 | 0.0 |
| B-core-3 | 19.8 | 0.0 | 0.0 | 0.0 | 48.0 | 41.0 | 0.0 | 15.8 | 70.1 | 13.6 | 0.0 | 0.0 | 0.0 |
| B-rind-1 | 57.9 | 0.0 | 0.0 | 0.0 | 181.1 | 0.0 | 0.0 | 10.2 | 197.1 | 0.0 | 0.0 | 0.0 | 0.0 |
| B-rind-2 | 28.4 | 0.0 | 0.0 | 0.0 | 31.1 | 61.2 | 0.0 | 4.6 | 59.2 | 0.0 | 0.0 | 0.0 | 0.0 |
| B-rind-3 | 363.1 | 209.6 | 0.0 | 70.7 | 184.8 | 50.5 | 13.2 | 16.9 | 371.0 | 46.2 | 0.0 | 235.5 | 0.0 |
| BM-core-1 | 157.6 | 0.0 | 0.0 | 14.6 | 98.0 | 13.7 | 0.0 | 13.9 | 368.0 | 23.9 | 0.0 | 0.0 | 25.0 |
| BM-core-2 | 191.8 | 0.0 | 10.3 | 21.8 | 140.3 | 16.3 | 0.0 | 44.1 | 589.7 | 80.4 | 0.0 | 0.0 | 59.8 |
| BM-core-3 | 85.7 | 26.6 | 11.2 | 11.0 | 0.0 | 40.9 | 0.0 | 0.0 | 18.5 | 0.0 | 0.0 | 0.0 | 0.0 |
| BM-rind-1 | 20.2 | 0.0 | 0.0 | 0.0 | 62.8 | 51.9 | 0.0 | 16.6 | 227.6 | 0.0 | 0.0 | 0.0 | 0.0 |
| BM-rind-2 | 30.1 | 0.0 | 0.0 | 0.0 | 71.1 | 100.1 | 0.0 | 24.7 | 291.7 | 28.4 | 0.0 | 0.0 | 0.0 |
| BM-rind-3 | 167.0 | 113.1 | 0.0 | 30.5 | 0.0 | 11.7 | 0.0 | 0.0 | 42.0 | 0.0 | 0.0 | 17.2 | 0.0 |
| C-core-1 | 137.0 | 78.8 | 0.0 | 38.7 | 92.7 | 217.4 | 6.8 | 25.6 | 86.7 | 16.6 | 0.0 | 0.0 | 0.0 |
| C-core-2 | 154.2 | 72.0 | 0.0 | 68.4 | 54.6 | 47.6 | 11.9 | 17.1 | 99.4 | 9.5 | 0.0 | 15.9 | 0.0 |
| C-core-3 | 18.7 | 0.0 | 0.0 | 0.0 | 20.2 | 232.1 | 0.0 | 9.6 | 74.7 | 0.0 | 0.0 | 0.0 | 0.0 |
| C-rind-1 | 20.2 | 0.0 | 0.0 | 0.0 | 13.0 | 288.1 | 0.0 | 15.9 | 33.1 | 0.0 | 0.0 | 0.0 | 0.0 |
| C-rind-2 | 16.4 | 0.0 | 0.0 | 0.0 | 18.3 | 140.3 | 0.0 | 12.1 | 59.1 | 0.0 | 0.0 | 0.0 | 0.0 |
| C-rind-3 | 93.2 | 42.0 | 0.0 | 59.6 | 26.3 | 20.6 | 8.5 | 5.9 | 59.2 | 0.0 | 0.0 | 0.0 | 0.0 |
| L-core-1 | 259.9 | 269.9 | 0.0 | 84.9 | 83.2 | 21.6 | 17.4 | 11.6 | 81.1 | 25.6 | 0.0 | 0.0 | 0.0 |
| L-core-2 | 186.1 | 105.1 | 0.0 | 32.6 | 146.7 | 74.1 | 0.0 | 29.4 | 194.4 | 37.7 | 0.0 | 18.5 | 0.0 |
| L-core-3 | 39.1 | 0.0 | 0.0 | 0.0 | 12.7 | 323.2 | 0.0 | 6.3 | 35.1 | 0.0 | 0.0 | 0.0 | 0.0 |
| L-rind-1 | 30.3 | 0.0 | 0.0 | 0.0 | 24.6 | 20.9 | 0.0 | 10.2 | 43.3 | 0.0 | 0.0 | 0.0 | 0.0 |
| L-rind-2 | 23.2 | 0.0 | 0.0 | 0.0 | 44.2 | 51.7 | 0.0 | 15.6 | 71.9 | 13.4 | 0.0 | 0.0 | 0.0 |
| L-rind-3 | 107.2 | 62.0 | 0.0 | 20.8 | 44.7 | 71.5 | 0.0 | 15.6 | 108.5 | 0.0 | 0.0 | 0.0 | 0.0 |
| M-core-1 | 269.0 | 155.1 | 0.0 | 47.0 | 30.5 | 26.6 | 0.0 | 0.0 | 29.5 | 0.0 | 0.0 | 52.2 | 0.0 |
| M-core-2 | 207.7 | 32.1 | 0.0 | 24.3 | 13.5 | 184.8 | 6.8 | 5.7 | 40.8 | 16.3 | 0.0 | 0.0 | 0.0 |
| M-core-3 | 33.7 | 0.0 | 0.0 | 0.0 | 104.6 | 90.6 | 0.0 | 0.0 | 133.0 | 15.5 | 13.0 | 0.0 | 49.1 |
| M-rind-1 | 27.9 | 0.0 | 0.0 | 0.0 | 18.9 | 135.7 | 0.0 | 7.9 | 25.7 | 0.0 | 0.0 | 0.0 | 0.0 |
| M-rind-2 | 38.7 | 0.0 | 0.0 | 0.0 | 6.2 | 257.0 | 0.0 | 5.8 | 77.8 | 0.0 | 0.0 | 0.0 | 0.0 |
| M-rind-3 | 271.0 | 0.0 | 26.4 | 50.2 | 169.3 | 13.9 | 8.7 | 12.8 | 806.9 | 62.4 | 33.6 | 14.5 | 115.6 |

Table S2. Free amino acids abundance, expressed as mg/%, in 30 cheese samples. B: Brie de Meaux; BM: Brie de Melun; C: Coulommiers; L: Langres; M:Maroilles

| Sample | Aspartic acid | Threonine | Serine | Glutamic acid | Proline | Glycine | Alanine | Cystine | Valine | Methionine | Isoleucine | Leucine | Tyrosine | Phenylalanine | GABA | Histidine | Tryptophan | Lysine | Arginine |
|---|---|---|---|---|---|---|---|---|---|---|---|---|---|---|---|---|---|---|---|
| B-core-1 | 0.284 | 0.090 | 0.065 | 1.858 | 0.925 | 0.432 | 0.709 | 0.014 | 0.922 | 0.245 | 0.630 | 1.436 | 0.227 | 0.745 | 0.011 | 0.166 | 0.266 | 0.317 | 1.321 |
| B-core-2 | 0.936 | 0.180 | 0.130 | 3.665 | 0.720 | 1.447 | 1.436 | 0.047 | 1.735 | 0.648 | 1.267 | 2.747 | 0.371 | 1.559 | 0.029 | 0.436 | 0.994 | 0.792 | 0.338 |
| B-core-3 | 0.065 | 0.025 | 0.029 | 0.569 | 0.212 | 0.155 | 0.216 | 0.004 | 0.310 | 0.047 | 0.194 | 0.486 | 0.097 | 0.266 | 0.133 | 0.065 | 0.094 | 0.086 | 0.281 |
| B-rind-1 | 1.004 | 0.313 | 0.425 | 5.627 | 4.702 | 1.332 | 3.762 | 0.094 | 2.563 | 0.770 | 1.660 | 3.370 | 1.303 | 1.782 | 0.072 | 1.123 | 0.378 | 0.371 | 1.667 |
| B-rind-2 | 1.343 | 0.817 | 0.349 | 5.018 | 1.256 | 1.534 | 2.437 | 0.076 | 1.775 | 1.127 | 1.343 | 2.473 | 1.048 | 1.613 | 0.040 | 1.156 | 0.569 | 0.749 | 0.295 |
| B-rind-3 | 0.500 | 0.122 | 0.101 | 5.281 | 1.994 | 0.727 | 3.744 | 0.072 | 1.580 | 0.288 | 1.051 | 1.951 | 0.662 | 0.979 | 0.050 | 0.832 | 0.248 | 0.212 | 0.349 |
| BM-core-1 | 1.076 | 0.396 | 0.040 | 1.930 | 0.626 | 0.400 | 0.662 | 0.007 | 0.734 | 0.342 | 0.551 | 1.235 | 0.036 | 0.767 | 0.036 | 0.335 | 0.972 | 1.134 | 1.138 |
| BM-core-2 | 0.702 | 0.238 | 0.133 | 2.765 | 0.392 | 0.576 | 0.922 | 0.104 | 1.076 | 0.601 | 0.713 | 1.966 | 0.281 | 1.206 | 0.040 | 0.392 | 0.842 | 0.518 | 0.205 |
| BM-core-3 | 0.641 | 0.436 | 0.436 | 1.840 | 0.475 | 0.418 | 0.616 | 0.007 | 0.752 | 0.252 | 0.493 | 0.972 | 0.374 | 0.623 | 0.032 | 0.572 | 0.180 | 0.734 | 0.119 |
| BM-rind-1 | 3.514 | 2.182 | 2.704 | 6.066 | 10.451 | 2.189 | 6.095 | 0.036 | 4.086 | 1.717 | 2.239 | 6.095 | 2.747 | 0.572 | 0.072 | 1.411 | 1.886 | 4.244 | 2.729 |
| BM-rind-2 | 1.508 | 0.796 | 1.577 | 2.873 | 3.283 | 0.860 | 2.146 | 0.040 | 1.264 | 0.828 | 0.644 | 2.243 | 1.177 | 0.781 | 0.068 | 1.076 | 1.037 | 1.422 | 0.313 |
| BM-rind-3 | 0.666 | 0.461 | 0.500 | 2.599 | 1.292 | 0.526 | 1.566 | 0.011 | 1.102 | 0.436 | 0.677 | 1.300 | 0.684 | 0.828 | 0.022 | 0.565 | 0.029 | 0.932 | 0.191 |
| C-core-1 | 0.083 | 0.022 | 0.011 | 0.594 | 0.299 | 0.144 | 0.241 | 0.000 | 0.295 | 0.140 | 0.194 | 0.626 | 0.065 | 0.252 | 0.022 | 0.148 | 0.194 | 0.086 | 1.238 |
| C-core-2 | 0.468 | 0.169 | 0.137 | 1.181 | 0.346 | 0.475 | 0.626 | 0.007 | 0.659 | 0.335 | 0.439 | 1.289 | 0.234 | 0.644 | 0.036 | 0.396 | 0.648 | 0.518 | 0.230 |
| C-core-3 | 0.133 | 0.029 | 0.025 | 0.619 | 0.130 | 0.065 | 0.122 | 0.000 | 0.184 | 0.083 | 0.104 | 0.421 | 0.083 | 0.324 | 0.025 | 0.173 | 0.079 | 0.072 | 0.378 |
| C-rind-1 | 0.378 | 0.407 | 0.025 | 1.537 | 0.918 | 0.310 | 1.224 | 0.032 | 0.468 | 0.191 | 0.259 | 0.572 | 0.176 | 0.043 | 0.018 | 0.317 | 0.094 | 0.094 | 0.392 |
| C-rind-2 | 0.504 | 0.680 | 0.043 | 4.201 | 2.812 | 1.156 | 4.298 | 0.097 | 1.696 | 0.745 | 1.004 | 2.538 | 0.572 | 0.198 | 0.068 | 1.454 | 0.504 | 0.677 | 0.079 |
| C-rind-3 | 0.317 | 0.475 | 0.202 | 1.166 | 0.490 | 0.263 | 1.822 | 0.011 | 0.371 | 0.108 | 0.184 | 0.385 | 0.144 | 0.043 | 0.014 | 0.295 | 0.083 | 0.320 | 0.148 |
| L-core-1 | 0.101 | 0.709 | 0.223 | 2.430 | 1.249 | 0.616 | 0.803 | 0.000 | 1.030 | 0.479 | 0.594 | 1.552 | 0.767 | 1.127 | 0.083 | 0.522 | 0.698 | 1.282 | 1.188 |
| L-core-2 | 0.176 | 1.606 | 0.353 | 10.408 | 4.435 | 2.099 | 2.873 | 0.018 | 3.834 | 1.476 | 2.020 | 4.777 | 1.033 | 0.173 | 0.058 | 1.634 | 1.670 | 3.744 | 0.292 |
| L-core-3 | 0.205 | 0.317 | 0.220 | 1.386 | 0.144 | 0.320 | 0.356 | 0.000 | 0.335 | 0.245 | 0.198 | 0.648 | 0.414 | 0.234 | 0.058 | 0.346 | 0.248 | 0.338 | 0.274 |
| L-rind-1 | 0.414 | 1.213 | 0.515 | 6.502 | 8.345 | 1.037 | 3.092 | 0.007 | 2.225 | 1.292 | 1.512 | 3.290 | 1.724 | 0.238 | 0.094 | 1.811 | 1.123 | 3.013 | 0.364 |
| L-rind-2 | 0.004 | 0.011 | 0.004 | 0.061 | 0.047 | 0.011 | 0.022 | 0.000 | 0.022 | 0.007 | 0.011 | 0.025 | 0.000 | 0.047 | 0.000 | 0.050 | 0.007 | 0.025 | 0.133 |
| L-rind-3 | 0.439 | 0.918 | 0.522 | 3.636 | 2.628 | 0.817 | 1.843 | 0.000 | 1.343 | 0.727 | 0.904 | 1.904 | 1.246 | 0.130 | 0.032 | 1.328 | 0.778 | 1.786 | 0.014 |
| M-core-1 | 0.961 | 0.184 | 0.162 | 1.670 | 0.223 | 0.396 | 0.796 | 0.058 | 1.130 | 0.317 | 0.526 | 1.462 | 0.338 | 0.904 | 0.040 | 0.331 | 0.680 | 1.076 | 0.266 |
| M-core-2 | 0.054 | 0.688 | 0.335 | 1.778 | 0.544 | 0.522 | 0.839 | 0.011 | 1.627 | 0.673 | 0.706 | 1.807 | 0.590 | 1.184 | 0.036 | 0.724 | 0.961 | 2.300 | 0.364 |
| M-core-3 | 0.086 | 0.310 | 0.133 | 1.840 | 0.180 | 0.515 | 0.994 | 0.022 | 1.368 | 0.569 | 0.648 | 1.894 | 0.274 | 1.116 | 0.068 | 0.029 | 0.756 | 1.627 | 0.137 |
| M-rind-1 | 0.277 | 0.122 | 0.277 | 5.407 | 0.216 | 1.483 | 3.373 | 0.284 | 3.460 | 1.030 | 1.350 | 2.707 | 1.512 | 1.505 | 0.104 | 0.266 | 1.177 | 2.650 | 0.256 |
| M-rind-2 | 0.248 | 0.990 | 0.634 | 4.190 | 0.911 | 0.932 | 2.408 | 0.025 | 2.653 | 1.094 | 1.224 | 3.179 | 1.156 | 1.937 | 0.086 | 0.569 | 1.472 | 3.287 | 0.292 |
| M-rind-3 | 0.166 | 0.562 | 0.371 | 3.780 | 0.025 | 0.720 | 1.735 | 0.014 | 1.732 | 0.709 | 0.918 | 2.045 | 0.720 | 0.065 | 0.068 | 0.194 | 0.821 | 2.164 | 0.317 |

Table S3. Volatile compounds detected in cheese samples by HS-GC/MS.

| No | Name | category |
|---|---|---|
| 1 | Methanethiol | sulfur compound |
| 2 | Dimethyl sulfide | sulfur compound |
| 3 | Propanal | aldehyde |
| 4 | Acetone | ketone |
| 5 | Ethyl acetate | ester |
| 6 | 2-Butanone | ketone |
| 7 | 3-Methylbutanal | aldehyde |
| 8 | 2-Propanol | alcohol |
| 9 | Ethanol | alcohol |
| 10 | 2-Pentanone | ketone |
| 11 | Methyl butanoate | ester |
| 12 | 3-Methylpentan-2-one | ketone |
| 13 | 2-Butanol | alcohol |
| 14 | Ethyl butanoate | ester |
| 15 | Methyl thiolacetate | sulfur compound |
| 16 | 2,3-Pentanedione | ketone |
| 17 | Pentan-2-yl acetate | ester |
| 18 | Dimethyl disulfide | sulfur compound |
| 19 | 2-Hexanone | ketone |
| 20 | 2-Methylpropan-1-ol | alcohol |
| 21 | 2-Propen-1-ol | alcohol |
| 22 | 2-Pentanol | alcohol |
| 23 | 3-Methylbutyl acetate | ester |
| 24 | Pentyl butanoate | ester |
| 25 | Propyl butanoate | ester |
| 26 | Pentan-2-yl propanoate | ester |
| 27 | *S*-Methyl butanethioate | sulfur compound |
| 28 | 1-Nonene | hydrocarbon |
| 29 | 2-Methylpropyl butanoate | ester |
| 30 | 2-Heptanone | ketone |
| 31 | 3-Methylbutyl propanoate | ester |
| 32 | 3-Methyl-1-butanol | alcohol |
| 33 | 2-Hexanol | alcohol |
| 34 | *S*-Methyl 3-methylbutanethioate | sulfur compound |
| 35 | Ethyl hexanoate | ester |
| 36 | 6-Methylhepan-2-one | ketone |
| 37 | 3-Hydroxy-3-methylbutan-2-one | ketone |
| 38 | 3-Methylbutyl butanoate | ester |
| 39 | 2-Octanone | ketone |
| 40 | Acetoin | ketone |
| 41 | 2,4-Dithiapentane | sulfur compound |
| 42 | 2-Heptanol | alcohol |
| 43 | Propyl hexanoate | ester |
| 44 | 2,5-Dimethylpyrazine | pyrazine |
| 45 | 2,6-Dimethylpytazine | pyrazine |
| 46 | 2-Hydroxy-2-methylpropanoic acid | carboxylic acid |
| 47 | 2-Hydroxypentan-3-one | ketone |
| 48 | 2-Ethylhexan-1-ol | alcohol |
| 49 | 2-Nonanone | ketone |
| 50 | Dimethyl trisulfide | sulfur compound |
| 51 | *S*-Methyl hexanethioate | sulfur compound |
| 52 | 2,3,5-Trimethylpyrazine | pyrazine |
| 53 | Ethyl octanoate | ester |
| 54 | 8-Nonen-2-one | ketone |
| 55 | 3-Methylbutyl hexanoate | ester |
| 56 | Methyl 2-hydroxy-4-methylpemtanoate | ester |
| 57 | 2-Decanone | ketone |
| 58 | 2-Nonanol | alcohol |

Table S4. Volatile compounds data (retension time, peak erea) in surface mold-ripened cheeses and bacterial smear-ripened cheeses. B: Brie de Meaux; BM: Brie de Melun; C: Coulommiers; L: Langres; M:Maroilles

| No | Name | Retention_Time (min) | B-core-1 | B-core-2 | B-core-3 | B-rind-1 | B-rind-2 | B-rind-3 | BM-core-1 | BM-core-2 | BM-core-3 | BM-rind-1 | BM-rind-2 | BM-rind-3 | C-core-1 | C-core-2 | C-core-3 | C-rind-1 |
|---|---|---|---|---|---|---|---|---|---|---|---|---|---|---|---|---|---|---|
| 1 | Methanethiol | 5.36 | 0 | 0 | 0 | 0 | 0 | 135530 | 0 | 0 | 0 | 0 | 0 | 0 | 0 | 0 | 0 | 99213 |
| 2 | Dimethyl sulfide | 5.87 | 436083 | 0 | 5858996 | 147705 | 0 | 853257 | 58525 | 142547 | 0 | 0 | 0 | 137486 | 244555 | 84834 | 3758648 | 170816 |
| 3 | Propanal | 6.20 | 0 | 0 | 0 | 0 | 0 | 0 | 0 | 0 | 0 | 0 | 0 | 0 | 0 | 0 | 0 | 0 |
| 4 | Acetone | 6.49 | 11468548 | 4215385 | 7587692 | 28613163 | 17612213 | 26953758 | 7963270 | 5520938 | 2286498 | 26770080 | 19461219 | 9608800 | 3242769 | 5712281 | 15490432 | 14511022 |
| 5 | Ethyl acetate | 7.51 | 0 | 0 | 0 | 0 | 0 | 0 | 0 | 0 | 0 | 0 | 0 | 0 | 0 | 0 | 0 | 0 |
| 6 | 2-Butanone | 7.77 | 11196515 | 1488499 | 2876947 | 35023706 | 3573098 | 5247834 | 841221 | 403883 | 738842 | 3975365 | 1677228 | 2569365 | 3115285 | 968632 | 7477403 | 9242906 |
| 7 | 3-Methylbutanal | 8.11 | 1506786 | 924792 | 1016871 | 8210393 | 1188668 | 1613078 | 225335 | 689986 | 772647 | 866539 | 1578614 | 676334 | 1777937 | 1652922 | 1332242 | 5704479 |
| 8 | 2-Propanol | 8.28 | 11904964 | 5678551 | 14844804 | 2947695 | 1977352 | 11878243 | 3215112 | 3650083 | 99223 | 1871723 | 3504451 | 0 | 9874228 | 4176346 | 2765812 | 4863034 |
| 9 | Ethanol | 8.44 | 1320244 | 1624632 | 1867268 | 0 | 0 | 0 | 0 | 1895334 | 0 | 0 | 0 | 0 | 2905879 | 1714611 | 0 | 3011343 |
| 10 | 2-Pentanone | 9.35 | 1735035 | 2100848 | 10130432 | 2821140 | 3992256 | 8403848 | 3133483 | 5221545 | 1195143 | 3185396 | 6714299 | 2069770 | 4005841 | 4052212 | 5976604 | 3546138 |
| 11 | Methyl butanoate | 9.49 | 0 | 0 | 0 | 0 | 0 | 0 | 0 | 0 | 0 | 0 | 0 | 0 | 0 | 0 | 0 | 0 |
| 12 | 3-Methylpentan-2-one | 10.27 | 0 | 0 | 0 | 0 | 202045 | 0 | 0 | 0 | 77197 | 135374 | 0 | 485474 | 0 | 0 | 0 | 0 |
| 13 | 2-Butanol | 10.37 | 7990405 | 1055729 | 2910436 | 7689831 | 0 | 2533175 | 155677 | 133787 | 0 | 0 | 0 | 0 | 4759025 | 386121 | 540827 | 2128590 |
| 14 | Ethyl butanoate | 10.66 | 0 | 0 | 0 | 0 | 0 | 0 | 0 | 0 | 0 | 0 | 0 | 0 | 0 | 0 | 0 | 0 |
| 15 | Methyl thiolacetate | 11.03 | 0 | 148851 | 281983 | 0 | 0 | 247699 | 0 | 0 | 0 | 0 | 0 | 0 | 129618 | 0 | 189270 | 0 |
| 16 | 2,3-Pentanedione | 11.10 | 0 | 0 | 0 | 0 | 0 | 0 | 0 | 0 | 0 | 0 | 110887 | 0 | 0 | 0 | 0 | 0 |
| 17 | Pentan-2-yl acetate | 11.54 | 0 | 0 | 0 | 0 | 0 | 240114 | 0 | 0 | 0 | 0 | 0 | 0 | 81245 | 0 | 0 | 0 |
| 18 | Dimethyl disulfide | 11.69 | 4444410 | 4408994 | 23801237 | 10234846 | 6586002 | 16097271 | 336627 | 0 | 385360 | 4756528 | 1467593 | 3807997 | 6080167 | 483625 | 19871925 | 7387421 |
| 19 | 2-Hexanone | 11.73 | 0 | 0 | 0 | 0 | 0 | 0 | 168858 | 190648 | 0 | 0 | 0 | 0 | 0 | 0 | 0 | 0 |
| 20 | 2-Methylpropan-1-ol | 11.89 | 211758 | 0 | 140459 | 790242 | 0 | 487857 | 0 | 0 | 0 | 0 | 94900 | 0 | 470150 | 0 | 0 | 149327 |
| 21 | 2-Propen-1-ol | 12.34 | 0 | 0 | 0 | 0 | 0 | 0 | 0 | 0 | 0 | 0 | 0 | 0 | 0 | 0 | 0 | 0 |
| 22 | 2-Pentanol | 12.54 | 813138 | 1048930 | 6641472 | 503270 | 500252 | 4240404 | 308280 | 802584 | 0 | 210876 | 1373150 | 0 | 4397357 | 1284572 | 428819 | 1168211 |
| 23 | 3-Methylbutyl acetate | 12.70 | 154820 | 0 | 0 | 183597 | 0 | 1033373 | 0 | 0 | 0 | 0 | 0 | 0 | 132759 | 0 | 0 | 0 |
| 24 | Pentyl butanoate | 12.70 | 0 | 0 | 0 | 0 | 0 | 0 | 0 | 0 | 0 | 0 | 0 | 0 | 0 | 0 | 0 | 0 |
| 25 | Propyl butanoate | 12.72 | 0 | 0 | 0 | 0 | 0 | 0 | 0 | 0 | 0 | 0 | 0 | 0 | 0 | 0 | 0 | 0 |
| 26 | Pentan-2-yl propanoate | 12.97 | 0 | 0 | 0 | 0 | 0 | 46290 | 0 | 0 | 0 | 0 | 0 | 0 | 0 | 0 | 0 | 0 |
| 27 | *S*-Methyl butanethioate | 13.05 | 0 | 0 | 0 | 0 | 0 | 0 | 0 | 0 | 0 | 0 | 0 | 0 | 0 | 0 | 0 | 0 |
| 28 | 1-Nonene | 13.13 | 0 | 0 | 0 | 0 | 0 | 0 | 0 | 0 | 0 | 0 | 0 | 0 | 0 | 0 | 0 | 0 |
| 29 | 2-Methylpropyl butanoate | 13.50 | 0 | 0 | 0 | 0 | 0 | 0 | 0 | 0 | 0 | 0 | 0 | 0 | 0 | 0 | 0 | 0 |
| 30 | 2-Heptanone | 14.06 | 1011808 | 305047 | 4068747 | 1278145 | 1490292 | 6234738 | 5115273 | 4821731 | 975008 | 3786510 | 17449940 | 563197 | 1212546 | 1980678 | 1505630 | 1842745 |
| 31 | 3-Methylbutyl propanoate | 14.18 | 0 | 0 | 0 | 0 | 0 | 192034 | 0 | 0 | 0 | 0 | 0 | 0 | 0 | 0 | 0 | 0 |
| 32 | 3-Methyl-1-butanol | 14.48 | 4429822 | 341703 | 6907677 | 8637386 | 926179 | 24622739 | 365262 | 316841 | 501290 | 390033 | 1876819 | 141062 | 6191282 | 731234 | 696418 | 5450641 |
| 33 | 2-Hexanol | 14.72 | 0 | 0 | 93066 | 0 | 0 | 0 | 0 | 0 | 0 | 0 | 0 | 0 | 0 | 0 | 0 | 0 |
| 34 | *S*-Methyl 3-methylbutanethioate | 15.06 | 0 | 0 | 0 | 0 | 0 | 0 | 0 | 0 | 0 | 0 | 0 | 0 | 0 | 0 | 0 | 0 |
| 35 | Ethyl hexanoate | 15.09 | 0 | 0 | 0 | 0 | 0 | 0 | 0 | 0 | 0 | 0 | 0 | 0 | 0 | 0 | 0 | 0 |
| 36 | 6-Methylhepan-2-one | 15.22 | 0 | 0 | 0 | 76194 | 0 | 65320 | 0 | 0 | 0 | 0 | 0 | 0 | 0 | 0 | 0 | 0 |
| 37 | 3-Hydroxy-3-methylbutan-2-one | 15.36 | 0 | 0 | 0 | 0 | 0 | 0 | 0 | 0 | 0 | 0 | 0 | 0 | 0 | 0 | 0 | 0 |
| 38 | 3-Methylbutyl butanoate | 15.75 | 32969 | 0 | 0 | 0 | 0 | 0 | 0 | 0 | 0 | 0 | 0 | 0 | 0 | 0 | 0 | 0 |
| 39 | 2-Octanone | 16.17 | 0 | 0 | 0 | 0 | 0 | 195434 | 0 | 0 | 0 | 287047 | 668464 | 0 | 0 | 0 | 0 | 0 |
| 40 | Acetoin | 16.25 | 638238 | 171406 | 1851521 | 632027 | 1788359 | 4113898 | 2089442 | 251840 | 293488 | 1522870 | 321524 | 126143 | 745198 | 1230804 | 6805970 | 3888303 |
| 41 | 2,4-Dithiapentane | 16.48 | 214766 | 0 | 765529 | 148870 | 0 | 471935 | 0 | 0 | 0 | 0 | 0 | 0 | 0 | 0 | 644644 | 105925 |
| 42 | 2-Heptanol | 16.63 | 194559 | 78035 | 2283617 | 0 | 93215 | 1758486 | 413535 | 829454 | 0 | 201793 | 2669839 | 0 | 1242419 | 481712 | 190131 | 199244 |
| 43 | Propyl hexanoate | 16.77 | 0 | 0 | 0 | 0 | 0 | 0 | 0 | 0 | 0 | 0 | 0 | 0 | 0 | 0 | 0 | 0 |
| 44 | 2,5-Dimethylpyrazine | 17.03 | 0 | 0 | 0 | 68474 | 0 | 90389 | 82480 | 0 | 0 | 140201 | 186160 | 86248 | 0 | 0 | 0 | 0 |
| 45 | 2,6-Dimethylpytazine | 17.14 | 0 | 0 | 0 | 0 | 98074 | 0 | 0 | 0 | 0 | 0 | 0 | 0 | 0 | 0 | 0 | 0 |
| 46 | 2-Hydroxy-2-methylpropanoic acid | 17.37 | 0 | 0 | 0 | 0 | 0 | 429276 | 0 | 0 | 0 | 0 | 0 | 0 | 0 | 0 | 0 | 0 |
| 47 | 2-Hydroxypentan-3-one | 17.62 | 0 | 0 | 81213 | 0 | 0 | 360977 | 0 | 0 | 0 | 94994 | 0 | 0 | 0 | 0 | 161951 | 96431 |
| 48 | 2-Ethylhexan-1-ol | 17.75 | 0 | 0 | 0 | 0 | 0 | 0 | 0 | 0 | 0 | 0 | 0 | 0 | 0 | 0 | 0 | 0 |
| 49 | 2-Nonanone | 18.07 | 504574 | 0 | 507703 | 588934 | 1239221 | 10288567 | 2863009 | 1356247 | 348786 | 11778774 | 24806266 | 4076045 | 163972 | 426275 | 255449 | 1823678 |
| 50 | Dimethyl trisulfide | 18.29 | 237104 | 83509 | 254636 | 622611 | 189014 | 664256 | 0 | 0 | 0 | 131475 | 0 | 112960 | 0 | 0 | 323848 | 0 |
| 51 | *S*-Methyl hexanethioate | 18.42 | 0 | 0 | 0 | 0 | 0 | 0 | 0 | 0 | 0 | 0 | 0 | 0 | 0 | 0 | 0 | 0 |
| 52 | 2,3,5-Trimethylpyrazine | 18.50 | 0 | 0 | 0 | 0 | 0 | 0 | 0 | 0 | 0 | 0 | 0 | 0 | 0 | 0 | 0 | 0 |
| 53 | Ethyl octanoate | 18.77 | 0 | 0 | 0 | 0 | 0 | 0 | 0 | 0 | 0 | 0 | 0 | 0 | 0 | 0 | 0 | 0 |
| 54 | 8-Nonen-2-one | 18.98 | 0 | 0 | 153705 | 0 | 125954 | 975725 | 408124 | 187936 | 0 | 884428 | 2577437 | 144451 | 0 | 0 | 0 | 226304 |
| 55 | 3-Methylbutyl hexanoate | 19.16 | 0 | 0 | 0 | 0 | 0 | 0 | 0 | 0 | 0 | 0 | 0 | 0 | 0 | 0 | 0 | 0 |
| 56 | Methyl 2-hydroxy-4-methylpemtanoate | 19.58 | 0 | 0 | 0 | 0 | 0 | 0 | 0 | 0 | 0 | 0 | 0 | 0 | 0 | 0 | 128715 | 0 |
| 57 | 2-Decanone | 19.78 | 0 | 0 | 0 | 0 | 0 | 0 | 0 | 0 | 0 | 141128 | 163710 | 111220 | 0 | 0 | 0 | 0 |
| 58 | 2-Nonanol | 19.94 | 0 | 0 | 0 | 0 | 0 | 854029 | 0 | 0 | 0 | 628559 | 998088 | 0 | 193951 | 0 | 0 | 0 |

| C-rind-2 | C-rind-3 | L-core-1 | L-core-2 | L-core-3 | L-rind-1 | L-rind-2 | L-rind-3 | M-core-1 | M-core-2 | M-core-3 | M-rind-1 | M-rind-2 | M-rind-3 |
|---|---|---|---|---|---|---|---|---|---|---|---|---|---|
| 0 | 157739 | 0 | 0 | 0 | 0 | 0 | 0 | 91493 | 0 | 0 | 0 | 254028 | 0 |
| 0 | 2618955 | 0 | 70195 | 1010792 | 0 | 0 | 991784 | 57514 | 0 | 107476 | 0 | 944926 | 145919 |
| 0 | 0 | 0 | 0 | 0 | 78470 | 0 | 65909 | 0 | 0 | 0 | 0 | 0 | 0 |
| 14357702 | 22263375 | 2649980 | 2682878 | 3768299 | 2838833 | 23740597 | 3742456 | 4115155 | 4469846 | 7680184 | 4126816 | 26792030 | 11226145 |
| 0 | 0 | 0 | 68220 | 237791 | 0 | 0 | 377843 | 0 | 0 | 0 | 0 | 0 | 0 |
| 2050976 | 17466680 | 199518 | 76475 | 209699 | 163724 | 543878 | 179395 | 31439306 | 3888970 | 5533964 | 33184578 | 25198869 | 5780978 |
| 2047279 | 1307189 | 442796 | 3471528 | 8303543 | 2187312 | 2639124 | 10026622 | 256025 | 1002982 | 1016737 | 2466086 | 4697875 | 11099542 |
| 3002913 | 5063305 | 9594099 | 6324975 | 10092523 | 11132121 | 3364673 | 12562741 | 247631 | 534880 | 361728 | 429541 | 165938 | 434539 |
| 0 | 0 | 4307567 | 12426462 | 11267341 | 6109236 | 0 | 17405136 | 0 | 0 | 0 | 0 | 0 | 1662976 |
| 5238850 | 8565659 | 316652 | 234187 | 296281 | 406351 | 1051828 | 422571 | 534641 | 650074 | 1055728 | 332135 | 2056504 | 986849 |
| 0 | 0 | 82804 | 175266 | 0 | 0 | 0 | 0 | 0 | 0 | 0 | 0 | 0 | 0 |
| 179707 | 0 | 0 | 0 | 0 | 0 | 0 | 0 | 0 | 0 | 0 | 0 | 0 | 0 |
| 0 | 3248579 | 392075 | 140834 | 289035 | 322904 | 183606 | 321212 | 0 | 293715 | 254787 | 1600374 | 0 | 219255 |
| 0 | 0 | 489599 | 2160856 | 0 | 0 | 0 | 0 | 0 | 0 | 0 | 0 | 0 | 0 |
| 0 | 481573 | 0 | 0 | 1440554 | 0 | 0 | 890564 | 0 | 0 | 0 | 0 | 0 | 0 |
| 0 | 0 | 0 | 0 | 0 | 0 | 0 | 0 | 0 | 0 | 0 | 0 | 0 | 0 |
| 0 | 0 | 0 | 0 | 0 | 0 | 0 | 0 | 0 | 0 | 0 | 0 | 0 | 0 |
| 7216321 | 28796962 | 549325 | 1260326 | 23770451 | 128856 | 582959 | 14602082 | 7482451 | 384383 | 5625252 | 6508285 | 25643484 | 9199927 |
| 0 | 0 | 0 | 0 | 0 | 0 | 0 | 0 | 0 | 0 | 0 | 0 | 0 | 0 |
| 0 | 137096 | 0 | 678235 | 1289534 | 0 | 302233 | 528687 | 0 | 0 | 141089 | 152065 | 313148 | 907437 |
| 0 | 0 | 0 | 0 | 0 | 95649 | 0 | 0 | 0 | 0 | 0 | 0 | 0 | 0 |
| 331172 | 622357 | 335176 | 166484 | 160892 | 296241 | 113412 | 134978 | 0 | 0 | 0 | 0 | 0 | 0 |
| 0 | 0 | 0 | 0 | 0 | 0 | 0 | 0 | 0 | 0 | 0 | 0 | 0 | 0 |
| 0 | 0 | 0 | 100156 | 1078795 | 0 | 0 | 0 | 0 | 0 | 0 | 0 | 0 | 0 |
| 0 | 0 | 0 | 0 | 0 | 0 | 0 | 627828 | 0 | 0 | 0 | 0 | 0 | 0 |
| 0 | 0 | 0 | 0 | 0 | 0 | 0 | 0 | 0 | 0 | 0 | 0 | 0 | 0 |
| 0 | 0 | 0 | 0 | 0 | 0 | 0 | 0 | 163403 | 0 | 0 | 0 | 0 | 0 |
| 0 | 0 | 0 | 0 | 0 | 0 | 0 | 141100 | 0 | 0 | 0 | 0 | 0 | 0 |
| 0 | 0 | 0 | 47722 | 0 | 0 | 0 | 0 | 0 | 0 | 0 | 0 | 0 | 0 |
| 2308193 | 5390069 | 335177 | 372364 | 461459 | 349744 | 1282295 | 636693 | 107853 | 134886 | 332414 | 87580 | 489825 | 436448 |
| 0 | 0 | 0 | 0 | 0 | 0 | 0 | 0 | 0 | 0 | 0 | 0 | 0 | 0 |
| 1981134 | 2105786 | 195383 | 2895115 | 13030687 | 546513 | 4059236 | 6121262 | 388015 | 495558 | 3493291 | 2335749 | 8466070 | 14245495 |
| 0 | 0 | 0 | 0 | 0 | 0 | 0 | 0 | 0 | 0 | 0 | 0 | 0 | 0 |
| 0 | 0 | 0 | 0 | 0 | 0 | 0 | 0 | 610741 | 0 | 0 | 149759 | 0 | 0 |
| 0 | 0 | 172928 | 384320 | 490908 | 175553 | 126826 | 784930 | 0 | 0 | 0 | 0 | 0 | 0 |
| 0 | 0 | 0 | 0 | 0 | 0 | 0 | 0 | 0 | 0 | 0 | 0 | 0 | 0 |
| 0 | 347026 | 0 | 0 | 0 | 0 | 0 | 0 | 0 | 0 | 0 | 0 | 0 | 0 |
| 0 | 0 | 0 | 255293 | 704199 | 0 | 508580 | 535771 | 0 | 0 | 0 | 0 | 0 | 165944 |
| 0 | 0 | 0 | 0 | 0 | 0 | 0 | 0 | 0 | 0 | 0 | 0 | 0 | 0 |
| 918092 | 3060773 | 789933 | 1658779 | 1967517 | 2533187 | 916718 | 5717157 | 372916 | 1801815 | 0 | 0 | 123053 | 201206 |
| 111794 | 909301 | 0 | 0 | 111032 | 0 | 0 | 205886 | 85300 | 0 | 0 | 0 | 429691 | 62955 |
| 138226 | 150402 | 0 | 0 | 91349 | 86613 | 0 | 118419 | 0 | 0 | 0 | 0 | 0 | 0 |
| 0 | 0 | 0 | 0 | 245283 | 0 | 0 | 198073 | 0 | 0 | 0 | 0 | 0 | 0 |
| 0 | 63844 | 0 | 0 | 0 | 0 | 0 | 0 | 456384 | 133420 | 847865 | 127945 | 1099700 | 329997 |
| 0 | 0 | 0 | 0 | 0 | 0 | 0 | 49337 | 0 | 0 | 0 | 0 | 435486 | 800933 |
| 0 | 0 | 0 | 0 | 0 | 0 | 0 | 0 | 0 | 0 | 0 | 0 | 0 | 0 |
| 0 | 148153 | 0 | 0 | 0 | 0 | 0 | 86913 | 0 | 0 | 0 | 0 | 0 | 0 |
| 0 | 0 | 0 | 0 | 0 | 0 | 0 | 0 | 0 | 0 | 0 | 73711 | 0 | 0 |
| 1151695 | 2623094 | 0 | 0 | 0 | 0 | 586784 | 251057 | 0 | 0 | 62378 | 0 | 91964 | 106565 |
| 374401 | 1116893 | 0 | 0 | 296518 | 0 | 0 | 195854 | 0 | 0 | 97719 | 1224886 | 303723 | 137413 |
| 0 | 0 | 0 | 0 | 147000 | 0 | 0 | 0 | 0 | 0 | 0 | 0 | 0 | 0 |
| 0 | 0 | 0 | 0 | 0 | 0 | 0 | 0 | 0 | 0 | 99643 | 0 | 214877 | 0 |
| 0 | 0 | 0 | 0 | 0 | 0 | 0 | 131779 | 0 | 0 | 0 | 0 | 0 | 0 |
| 123987 | 323145 | 0 | 0 | 0 | 0 | 0 | 0 | 0 | 0 | 0 | 0 | 0 | 0 |
| 0 | 0 | 0 | 0 | 113168 | 0 | 166453 | 0 | 0 | 0 | 0 | 0 | 0 | 0 |
| 0 | 0 | 0 | 0 | 0 | 0 | 0 | 0 | 0 | 0 | 0 | 0 | 0 | 0 |
| 0 | 0 | 0 | 0 | 0 | 0 | 0 | 0 | 0 | 0 | 0 | 0 | 0 | 0 |
| 0 | 0 | 0 | 0 | 0 | 0 | 0 | 0 | 0 | 0 | 0 | 0 | 0 | 0 |

Table S5. Categorical data of the volatile compounds detected in 30 cheese samples . These compounds were classified into eight categories (alcohol, aldehyde, carboxylic acid, ester, hydrocarbon, ketone, pyrazine, and sulfur compound) and counted. B: Brie de Meaux; BM: Brie de Melun; C: Coulommiers; L: Langres; M:Maroilles

| Sample | alcohol | aldehyde | carboxylic acid | ester | hydrocarbon | ketone | pyrazine | sulfur compound |
|---|---|---|---|---|---|---|---|---|
| B_core_1 | 7 | 1 | 0 | 2 | 0 | 6 | 0 | 4 |
| B_core_2 | 6 | 1 | 0 | 0 | 0 | 5 | 0 | 3 |
| B_core_3 | 8 | 1 | 0 | 0 | 0 | 8 | 0 | 5 |
| B_rind_1 | 5 | 1 | 0 | 1 | 0 | 7 | 1 | 4 |
| B_rind_2 | 4 | 1 | 0 | 0 | 0 | 8 | 1 | 2 |
| B_rind_3 | 7 | 1 | 1 | 4 | 0 | 10 | 1 | 6 |
| BM_core_1 | 5 | 1 | 0 | 0 | 0 | 8 | 1 | 2 |
| BM_core_2 | 6 | 1 | 0 | 0 | 0 | 8 | 0 | 1 |
| BM_core_3 | 2 | 1 | 0 | 0 | 0 | 7 | 0 | 1 |
| BM_rind_1 | 5 | 1 | 0 | 0 | 0 | 11 | 1 | 2 |
| BM_rind_2 | 6 | 1 | 0 | 0 | 0 | 10 | 1 | 1 |
| BM_rind_3 | 1 | 1 | 0 | 0 | 0 | 9 | 1 | 3 |
| C_core_1 | 8 | 1 | 0 | 2 | 0 | 6 | 0 | 3 |
| C_core_2 | 6 | 1 | 0 | 0 | 0 | 6 | 0 | 2 |
| C_core_3 | 5 | 1 | 0 | 1 | 0 | 7 | 0 | 5 |
| C_rind_1 | 7 | 1 | 0 | 0 | 0 | 8 | 0 | 4 |
| C_rind_2 | 4 | 1 | 0 | 0 | 0 | 8 | 0 | 3 |
| C_rind_3 | 6 | 1 | 0 | 0 | 0 | 9 | 1 | 6 |
| L_core_1 | 5 | 1 | 0 | 3 | 0 | 5 | 0 | 1 |
| L_core_2 | 6 | 1 | 0 | 7 | 0 | 5 | 0 | 2 |
| L_core_3 | 7 | 1 | 0 | 6 | 0 | 5 | 0 | 6 |
| L_rind_1 | 7 | 2 | 0 | 1 | 0 | 5 | 0 | 1 |
| L_rind_2 | 5 | 1 | 0 | 3 | 0 | 6 | 0 | 1 |
| L_rind_3 | 7 | 2 | 0 | 6 | 1 | 7 | 1 | 5 |
| M_core_1 | 2 | 1 | 0 | 0 | 0 | 5 | 1 | 6 |
| M_core_2 | 3 | 1 | 0 | 0 | 0 | 5 | 1 | 1 |
| M_core_3 | 4 | 1 | 0 | 0 | 0 | 5 | 2 | 3 |
| M_rind_1 | 5 | 1 | 0 | 0 | 0 | 4 | 1 | 3 |
| M_rind_2 | 3 | 1 | 0 | 0 | 0 | 6 | 3 | 5 |
| M_rind_3 | 5 | 1 | 0 | 1 | 0 | 6 | 2 | 4 |

Table S6. Significant positive correlations between bacteria and organic acids in cheese samples.

| Cluster (Bacteria) | Phylum | Genus | Cluster (Compounds) | Compound | Correlation (ρ) |
|---|---|---|---|---|---|
| β | *Firmicutes* | *Pediococcus* | II | Formic acid | 0.428 |
| | | | II | Acetic acid | 0.379 |
| γ | *Actinobacteria* | *Corynebacterium 1* | III | Isobutyric acid | 0.575 |
| | *Bacteroidetes* | *Psychroflexus* | III | Isobutyric acid | 0.556 |
| | *Epsilonbacteraeota* | *Arcobacter* | III | Isobutyric acid | 0.619 |
| | | | III | 3-Methylbutanoic acid | 0.375 |
| | *Firmicutes* | *Alkalibacterium* | III | Isobutyric acid | 0.583 |
| | | *Marinilactibacillus* | III | Isobutyric acid | 0.543 |
| | | *Vagococcus* | III | Isobutyric acid | 0.455 |
| | | *Clostridiisalibacter* | III | Isobutyric acid | 0.678 |
| | | | III | 3-Methylbutanoic acid | 0.438 |
| | *Proteobacteria* | *Psychrobacter* | III | Isobutyric acid | 0.388 |

Table S7. Significant negative correlations between bacteria and organic acids in cheese samples.

| Cluster (Bacteria) | Phylum | Genus | Cluster (Compounds) | Compound | Correlation (ρ) |
|---|---|---|---|---|---|
| α | *Firmicutes* | *Leuconostoc* | III | Pyruvic acid | -0.417 |
| | | | III | 3-Methylbutanoic acid | -0.382 |
| | *Proteobacteria* | *Pseudomonas* | III | 3-Methylbutanoic acid | -0.381 |
| β | *Firmicutes* | *Pediococcus* | I | Citric acid | -0.369 |
| | | *Enterococcus* | I | Alpha-Ketogulutaric acid | -0.364 |
| γ | *Actinobacteria* | *Corynebacterium 1* | II | Formic acid | -0.427 |
| | *Epsilonbacteraeota* | *Arcobacter* | II | Formic acid | -0.417 |
| | *Firmicutes* | *Marinilactibacillus* | II | Formic acid | -0.422 |
| | | *Vagococcus* | II | Formic acid | -0.364 |
| | *Proteobacteria* | *Marinobacter* | II | Succinic acid | -0.426 |
| | | | II | Formic acid | -0.655 |
| | | | II | Acetic acid | -0.413 |
| | | *Psychrobacter* | II | Formic acid | -0.394 |
| δ | *Actinobacteria* | *Brevibacterium* | II | Formic acid | -0.471 |
| | | *Brachybacterium* | II | Formic acid | -0.383 |
| | | | I | Propionic acid | -0.472 |
| | | *Leucobacter* | I | Propionic acid | -0.391 |
| | *Proteobacteria* | *Halomonas* | II | Succinic acid | -0.404 |
| | | | II | Formic acid | -0.424 |

Table S8. Significant positive correlations between bacteria and free amino acids in cheese samples.

| Cluster (Bacteria) | Phylum | Genus | Cluster (Compounds) | Compound | Correlation (ρ) |
|---|---|---|---|---|---|
| α | *Actinobacteria* | *Corynebacterium 1* | II | Valine | 0.366 |
| | | | II | Tryptophan | 0.374 |
| | | | II | Lysine | 0.491 |
| | | *Brevibacterium* | II | Alanine | 0.395 |
| | | | II | Cystine | 0.474 |
| | | | II | Valine | 0.382 |
| | | *Glutamicibacter* | II | Cystine | 0.470 |
| | *Bacteroidetes* | *Psychroflexus* | II | Lysine | 0.367 |
| | *Epsilonbacteraeota* | *Arcobacter* | II | Valine | 0.363 |
| | | | II | Tryptophan | 0.365 |
| | | | II | Lysine | 0.484 |
| | *Firmicutes* | *Alkalibacterium* | II | Lysine | 0.391 |
| | | *Marinilactibacillus* | II | Valine | 0.437 |
| | | | II | Phenylalanine | 0.384 |
| | | | II | GABA | 0.400 |
| | | | II | Lysine | 0.434 |
| | | *Vagococcus* | II | Cystine | 0.444 |
| | | | II | Valine | 0.512 |
| | | | II | Isoleucine | 0.375 |
| | | | II | Leucine | 0.372 |
| | | | II | Phenylalanine | 0.378 |
| | | | II | GABA | 0.373 |
| | | | II | Lysine | 0.387 |
| | *Proteobacteria* | *Marinobacter* | II | Lysine | 0.400 |
| | | *Halomonas* | II | Lysine | 0.375 |
| | | *Psychrobacter* | II | Cystine | 0.634 |
| | | | II | Valine | 0.452 |
| | | | II | Phenylalanine | 0.480 |
| β | *Proteobacteria* | *Pseudoalteromonas* | II | Valine | 0.420 |
| | | | II | Methionine | 0.389 |
| | | | II | Tryptophan | 0.529 |
| | | | II | Lysine | 0.661 |
| | | *Marinomonas* | II | Glutamic acid | 0.365 |
| | | | II | Methionine | 0.376 |
| | | | II | Tyrosine | 0.382 |
| | | | II | Lysine | 0.386 |
| | | *Pseudomonas* | II | Threonine | 0.371 |
| | | | I | Histidine | 0.388 |
| | | *Vibrio* | II | Tyrosine | 0.362 |
| | | | II | Tryptophan | 0.396 |
| | | | II | Lysine | 0.532 |
| γ | *Actinobacteria* | *Brachybacterium* | I | Aspartic acid | 0.474 |
| | | | II | Alanine | 0.379 |
| | | | II | Cystine | 0.448 |
| | | *Leucobacter* | II | Alanine | 0.396 |
| | | | II | Cystine | 0.413 |
| | *Firmicutes* | *Pediococcus* | I | Aspartic acid | 0.539 |
| | | | II | Tryptophan | 0.370 |
| δ | *Firmicutes* | *Carnobacterium* | I | Aspartic acid | 0.402 |
| | | | I | Arginine | 0.394 |
| | | | II | Cystine | 0.419 |

Table S9. Significant negative correlations between bacteria and free amino acids in cheese samples.

| Cluster (Bacteria) | Phylum | Genus | Cluster (Compounds) | Compound | Correlation (ρ) |
|---|---|---|---|---|---|
| α | *Actinobacteria* | *Corynebacterium 1* | I | Proline | -0.386 |
| | *Epsilonbacteraeota* | *Arcobacter* | I | Proline | -0.415 |
| | *Firmicutes* | *Alkalibacterium* | I | Proline | -0.389 |
| | | *Clostridiisalibacter* | I | Proline | -0.463 |
| | | | I | Histidine | -0.401 |
| | *Proteobacteria* | *Halomonas* | I | Arginine | -0.364 |
| β | *Proteobacteria* | *Vibrio* | I | Aspartic acid | -0.408 |
| δ | *Firmicutes* | *Carnobacterium* | II | Lysine | -0.390 |
| | | *Enterococcus* | II | Threonine | -0.390 |
| | | | II | Serine | -0.439 |
| | | | II | Lysine | -0.448 |
| ε | *Firmicutes* | *Lactobacillus* | II | Valine | -0.377 |
| | | | II | Lysine | -0.414 |
| | | *Lactococcus* | II | Glutamic acid | -0.385 |
| | | | II | Alanine | -0.514 |
| | | | II | Valine | -0.480 |
| | | | II | Isoleucine | -0.390 |
| | *Proteobacteria* | *Hafnia-Obesumbacterium* | II | Threonine | -0.462 |
| | | | II | Serine | -0.396 |
| | | | II | Tryptophan | -0.371 |
| | | | II | Lysine | -0.558 |

Table S10. Significant positive correlations between bacteria and volatile compounds in cheese samples.

| Cluster (Bacteria) | Phylum | Genus | Cluster (Compounds) | Compound | Compound (class) | Correlation (ρ) |
|---|---|---|---|---|---|---|
| | *Proteobacteria* | *Pseudoalteromonas* | IV | 2,5-Dimethylpyrazine | pyrazine | 0.396 |
| | | *Providencia* | I | Methyl butanoate | ester | 0.500 |
| | | | I | Ethyl butanoate | ester | 0.500 |
| | | | I | Pentyl butanoate | ester | 0.463 |
| | | | I | 2-Methylpropyl butanoate | ester | 0.719 |
| | | | I | Ethyl hexanoate | ester | 0.488 |
| | | | I | 3-Methylbutyl butanoate | ester | 0.529 |
| | | | I | 3-Methylbutyl hexanoate | ester | 0.463 |
| | | *Marinomonas* | I | Propanal | aldehyde | 0.598 |
| | | | I | Ethyl Acetate | ester | 0.421 |
| | | | I | Ethanol | alcohol | 0.433 |
| | | | I | Methyl butanoate | ester | 0.532 |
| | | | I | Ethyl butanoate | ester | 0.532 |
| | | | I | 2-Propen-1-ol | alcohol | 0.445 |
| | | | I | Propyl butanoate | ester | 0.384 |
| | | | I | 1-Nonene | hydrocarbon | 0.384 |
| α | | | I | 2-Methylpropyl butanoate | ester | 0.414 |
| | | | I | Ethyl hexanoate | ester | 0.797 |
| | | | I | 3-Methylbutyl butanoate | ester | 0.410 |
| | | | I | Ethyl octanoate | ester | 0.384 |
| | | *Pseudomonas* | I | Propanal | aldehyde | 0.451 |
| | | | I | Ethyl Acetate | ester | 0.469 |
| | | | II | 2-Propanol | alcohol | 0.418 |
| | | | I | Methyl butanoate | ester | 0.369 |
| | | | I | Ethyl butanoate | ester | 0.369 |
| | | | I | Ethyl hexanoate | ester | 0.724 |
| | | | I | 3-Methylbutyl butanoate | ester | 0.390 |
| | | | I | Propyl hexanoate | ester | 0.365 |
| | | *Vibrio* | I | Propanal | aldehyde | 0.445 |
| | | | I | Ethyl Acetate | ester | 0.403 |
| | | | IV | 3-Methylbutanal | aldehyde | 0.442 |
| | | | I | Ethyl hexanoate | ester | 0.659 |
| | | | I | 3-Methylbutyl butanoate | ester | 0.487 |
| | *Actinobacteria* | *Brachybacterium* | III | Acetone | ketone | 0.390 |
| | | | IV | 2-Butanone | ketone | 0.390 |
| | | | III | 3-Methylpentan-2-one | ketone | 0.566 |
| | | | III | 2-Nonanone | ketone | 0.421 |
| | | | III | 8-Nonen-2-one | ketone | 0.453 |
| | | *Leucobacter* | III | 3-Hydroxy-3-methylbutan-2-one | ketone | 0.413 |
| | | | IV | Dimethyl trisulfide | sulfer | 0.409 |
| | *Firmicutes* | *Staphylococcus* | III | Acetone | ketone | 0.683 |
| | | | IV | 2-Butanone | ketone | 0.491 |
| | | | IV | 2,5-Dimethylpyrazine | pyrazine | 0.368 |
| | | | III | 2-Nonanone | ketone | 0.538 |
| | | | III | 8-Nonen-2-one | ketone | 0.466 |
| | | *Carnobacterium* | III | Acetone | ketone | 0.523 |
| | | | III | 2-Pentanone | ketone | 0.667 |
| | | | II | 2-Pentanol | alcohol | 0.699 |
| | | | III | 3-Methylbutyl acetate | ester | 0.376 |
| | | | III | 2-Heptanone | ketone | 0.574 |
| | | | III | 2-Octanone | ketone | 0.377 |
| | | | II | 2-Heptanol | alcohol | 0.565 |
| | | | III | 2-Nonanone | ketone | 0.584 |
| | | | III | 8-Nonen-2-one | ketone | 0.545 |
| | | | III | 2-Nonanol | alcohol | 0.420 |
| β | | *Enterococcus* | III | Acetone | ketone | 0.496 |
| | | | III | 2-Pentanone | ketone | 0.473 |
| | | | II | 2-Pentanol | alcohol | 0.519 |
| | | | III | 3-Methylbutyl acetate | ester | 0.471 |
| | | | IV | 3-Methyl-1-butanol | alcohol | 0.400 |
| | | | III | 6-Methylhepan-2-one | ketone | 0.371 |
| | | | IV | Dimethyl trisulfide | sulfer | 0.372 |
| | | *Pediococcus* | III | 2-Pentanone | ketone | 0.372 |
| | | | III | 2-Hexanone | ketone | 0.596 |
| | | | II | 2-Heptanone | ketone | 0.549 |
| | | | III | 2-Octanone | ketone | 0.506 |
| | | | II | 2-Heptanol | alcohol | 0.480 |
| | | | III | 2-Nonanone | ketone | 0.545 |
| | | | III | 8-Nonen-2-one | ketone | 0.626 |
| | | | III | 2-Decanone | ketone | 0.506 |
| | | | III | 2-Nonanol | alcohol | 0.404 |
| | *Proteobacteria* | *Hafnia-Obesumbacterium* | III | 2-Pentanone | ketone | 0.673 |

| | | | | | | |
|---|---|---|---|---|---|---|
| | | | III | Pentan-2-yl acetate | ester | 0.367 |
| | | | II | 2-Pentanol | alcohol | 0.558 |
| | | | III | 3-Methylbutyl acetate | ester | 0.538 |
| | | | III | 2-Heptanone | ketone | 0.465 |
| | | | III | 6-Methylhepan-2-one | ketone | 0.377 |
| | | | III | 2,4-Dithiapentane | sulfer | 0.390 |
| | | | II | 2-Heptanol | alcohol | 0.462 |
| | *Firmicutes* | *Lactobacillus* | III | 2-Pentanone | ketone | 0.456 |
| | | | II | 2-Pentanol | alcohol | 0.525 |
| | | | III | 2-Heptanone | ketone | 0.447 |
| | | | II | 2-Heptanol | alcohol | 0.496 |
| | | | III | 2-Nonanone | ketone | 0.426 |
| | | *Leuconostoc* | II | 2-Propanol | alcohol | 0.483 |
| γ | | | II | 2-Pentanol | alcohol | 0.377 |
| | | | III | 6-Methylhepan-2-one | ketone | 0.373 |
| | | | II | Acetoin | ketone | 0.470 |
| | | *Lactococcus* | II | 2-Propanol | alcohol | 0.387 |
| | | | I | Ethanol | alcohol | 0.370 |
| | | | II | 2-Pentanol | alcohol | 0.428 |
| | | | II | 2-Heptanol | alcohol | 0.458 |
| | *Actinobacteria* | *Corynebacterium 1* | IV | 2-Butanone | ketone | 0.489 |
| | | | IV | *S*-Methyl 3-methylbutanethioate | sulfer | 0.441 |
| | | | IV | 2,5-Dimethylpyrazine | pyrazine | 0.727 |
| | | | IV | 2,6-Dimethylpytazine | pyrazine | 0.392 |
| | | | IV | 2,3,5-Trimethylpyrazine | pyrazine | 0.598 |
| | | *Brevibacterium* | III | Acetone | ketone | 0.428 |
| | | | IV | 2-Butanone | ketone | 0.651 |
| | | | IV | 2,5-Dimethylpyrazine | pyrazine | 0.610 |
| | | *Glutamicibacter* | IV | Methanethiol | sulfer | 0.500 |
| | | | III | Acetone | ketone | 0.388 |
| | | | IV | 2-Butanone | ketone | 0.739 |
| | | | III | Dimethyl disulfide | sulfer | 0.612 |
| | | | IV | 2,5-Dimethylpyrazine | pyrazine | 0.447 |
| | | | III | Dimethyl trisulfide | sulfer | 0.458 |
| | *Bacteroidetes* | *Psychroflexus* | IV | 2-Butanone | ketone | 0.531 |
| | | | IV | *S*-Methyl 3-methylbutanethioate | sulfer | 0.417 |
| | | | IV | 2,5-Dimethylpyrazine | pyrazine | 0.657 |
| | | | IV | 2,3,5-Trimethylpyrazine | pyrazine | 0.540 |
| | *Epsilonbacteraeota* | *Arcobacter* | IV | 2-Butanone | ketone | 0.491 |
| | | | IV | *S*-Methyl 3-methylbutanethioate | sulfer | 0.484 |
| | | | IV | 2,5-Dimethylpyrazine | pyrazine | 0.719 |
| | | | IV | 2-Ethylhexan-1-ol | alcohol | 0.384 |
| | | | IV | 2,3,5-Trimethylpyrazine | pyrazine | 0.551 |
| δ | *Firmicutes* | *Alkalibacterium* | IV | 2-Butanone | ketone | 0.545 |
| | | | IV | *S*-Methyl 3-methylbutanethioate | sulfer | 0.477 |
| | | | IV | 2,5-Dimethylpyrazine | pyrazine | 0.651 |
| | | | IV | 2,3,5-Trimethylpyrazine | pyrazine | 0.519 |
| | | *Marinilactibacillus* | IV | 2-Butanone | ketone | 0.596 |
| | | | IV | *S*-Methyl 3-methylbutanethioate | sulfer | 0.477 |
| | | | IV | 2,5-Dimethylpyrazine | pyrazine | 0.732 |
| | | | IV | 2,3,5-Trimethylpyrazine | pyrazine | 0.522 |
| | | *Vagococcus* | IV | 2-Butanone | ketone | 0.509 |
| | | | IV | *S*-Methyl 3-methylbutanethioate | sulfer | 0.416 |
| | | | IV | 2,5-Dimethylpyrazine | pyrazine | 0.487 |
| | | | IV | 2,6-Dimethylpytazine | pyrazine | 0.416 |
| | | | IV | 2,3,5-Trimethylpyrazine | pyrazine | 0.419 |
| | *Proteobacteria* | *Clostridiisalibacter* | IV | *S*-Methyl 3-methylbutanethioate | sulfer | 0.369 |
| | | | IV | 2,5-Dimethylpyrazine | pyrazine | 0.457 |
| | | | IV | 2-Ethylhexan-1-ol | alcohol | 0.597 |
| | | *Halomonas* | IV | 2,5-Dimethylpyrazine | pyrazine | 0.450 |
| | | | IV | 2,3,5-Trimethylpyrazine | pyrazine | 0.380 |
| | | *Marinobacter* | IV | *S*-Methyl butanethioate | sulfer | 0.372 |
| | | | IV | *S*-Methyl 3-methylbutanethioate | sulfer | 0.468 |
| | | | IV | 2,5-Dimethylpyrazine | pyrazine | 0.609 |
| | | | IV | 2,3,5-Trimethylpyrazine | pyrazine | 0.398 |
| | | *Psychrobacter* | IV | 2-Butanone | ketone | 0.671 |
| | | | IV | *S*-Methyl 3-methylbutanethioate | sulfer | 0.417 |
| | | | IV | 2,5-Dimethylpyrazine | pyrazine | 0.685 |

Table S11. Significant negative correlations between bacteria and volatile compounds in cheese samples.

| Cluster (Bacteria) | Phylum | Genus | Cluster (compounds) | Compound | Compound (class) | Correlation (ρ) |
|---|---|---|---|---|---|---|
| | *Proteobacteria* | *Pseudoalteromonas* | III | 2-Pentanone | ketone | -0.652 |
| | | | III | Methyl thiolacetate | sulfer | -0.396 |
| | | | II | 2-Pentanol | alcohol | -0.655 |
| | | | II | 2-Heptanone | ketone | -0.542 |
| | | | II | 2-Heptanol | alcohol | -0.568 |
| | | | III | 2-Nonanone | ketone | -0.496 |
| | | | III | 8-Nonen-2-one | ketone | -0.434 |
| | | *Marinomonas* | III | Acetone | ketone | -0.424 |
| | | | IV | 2-Butanone | ketone | -0.496 |
| | | | III | 2-Pentanone | ketone | -0.613 |
| α | | | II | 2-Heptanone | ketone | -0.372 |
| | | | III | 2-Nonanone | ketone | -0.417 |
| | | | III | 8-Nonen-2-one | ketone | -0.365 |
| | | *Pseudomonas* | IV | 2-Butanone | ketone | -0.509 |
| | | | IV | 2,5-Dimethylpyrazine | pyrazine | -0.400 |
| | | *Vibrio* | III | 2-Pentanone | ketone | -0.793 |
| | | | III | 3-Methylpentan-2-one | ester | -0.369 |
| | | | II | 2-Pentanol | alcohol | -0.594 |
| | | | II | 2-Heptanone | ketone | -0.643 |
| | | | II | 2-Heptanol | alcohol | -0.597 |
| | | | III | 2-Nonanone | ketone | -0.639 |
| | | | III | 8-Nonen-2-one | ketone | -0.534 |
| | *Actinobacteria* | *Brachybacterium* | II | 2-Propanol | alcohol | -0.434 |
| | | | I | Ethanol | alcohol | -0.534 |
| | | | I | Ethyl hexanoate | ester | -0.533 |
| | | | I | 3-Methylbutyl butanoate | ester | -0.475 |
| | *Firmicutes* | *Staphylococcus* | I | Ethyl Acetate | ester | -0.399 |
| β | | | I | Ethanol | alcohol | -0.499 |
| | | | I | Ethyl hexanoate | ester | -0.458 |
| | | | I | Propyl hexanoate | ester | -0.402 |
| | | *Carnobacterium* | I | Ethyl hexanoate | ester | -0.435 |
| | | *Pediococcus* | III | 2-Butanol | alcohol | -0.458 |
| | *Proteobacteria* | *Hafnia-Obesumbacterium* | I | Ethyl hexanoate | ester | -0.533 |
| | *Firmicutes* | *Lactobacillus* | IV | 3-Methylbutanal | aldehyde | -0.463 |
| | | | IV | *S*-Methyl 3-methylbutanethioate | sulfer | -0.402 |
| | | | IV | 2,5-Dimethylpyrazine | pyrazine | -0.430 |
| | | *Leuconostoc* | IV | 2,5-Dimethylpyrazine | pyrazine | -0.366 |
| γ | | *Lactococcus* | IV | Methanethiol | sulfer | -0.411 |
| | | | III | Acetone | ketone | -0.441 |
| | | | IV | 2-Butanone | ketone | -0.507 |
| | | | IV | 2,5-Dimethylpyrazine | pyrazine | -0.466 |
| | | | IV | 2,6-Dimethylpyrazine | pyrazine | -0.413 |
| | *Actinobacteria* | *Corynebacterium 1* | II | 2-Propanol | alcohol | -0.575 |
| | | | III | 2-Pentanone | alcohol | -0.365 |
| | | | II | 2-Pentanol | alcohol | -0.636 |
| | | | III | 2-Heptanone | ketone | -0.570 |
| | | | II | Acetoin | ketone | -0.491 |
| | | | II | 2-Heptanol | alcohol | -0.533 |
| | | | III | 2-Nonanone | ketone | -0.461 |
| | | | III | 8-Nonen-2-one | ketone | -0.365 |
| | | *Brevibacterium* | II | 2-Propanol | alcohol | -0.595 |
| | | | I | Ethanol | alcohol | -0.574 |
| | | | I | Ethyl hexanoate | ester | -0.557 |
| | | | I | 3-Methylbutyl butanoate | ester | -0.397 |
| | | *Glutamicibacter* | I | Ethanol | alcohol | -0.367 |
| | | | I | Ethyl hexanoate | ester | -0.499 |
| | *Bacteroidetes* | *Psychroflexus* | II | 2-Propanol | alcohol | -0.532 |
| | | | II | 2-Pentanol | alcohol | -0.551 |
| | | | III | 2-Heptanone | ketone | -0.520 |
| | | | II | Acetoin | ketone | -0.389 |
| | | | II | 2-Heptanol | alcohol | -0.471 |
| | | | III | 2-Nonanone | ketone | -0.387 |
| | *Epsilonbacteraeota* | *Arcobacter* | II | 2-Propanol | alcohol | -0.570 |
| | | | III | 2-Pentanone | ketone | -0.379 |
| | | | II | 2-Pentanol | alcohol | -0.636 |
| | | | III | 2-Heptanone | ketone | -0.582 |

| | | | | | | |
|---|---|---|---|---|---|---|
| | | | II | Acetoin | ketone | -0.503 |
| | | | II | 2-Heptanol | alcohol | -0.533 |
| | | | III | 2-Nonanone | ketone | -0.464 |
| | | | III | 8-Nonen-2-one | ketone | -0.365 |
| | *Firmicutes* | *Alkalibacterium* | II | 2-Propanol | alcohol | -0.533 |
| | | | II | 2-Pentanol | alcohol | -0.551 |
| | | | III | 2-Heptanone | ketone | -0.528 |
| | | | II | Acetoin | ketone | -0.413 |
| | | | II | 2-Heptanol | alcohol | -0.471 |
| | | | III | 2-Nonanone | ketone | -0.391 |
| | | *Marinilactibacillus* | II | 2-Propanol | alcohol | -0.585 |
| | | | II | 2-Pentanol | alcohol | -0.587 |
| | | | II | 2-Heptanone | ketone | -0.550 |
| | | | II | Acetoin | ketone | -0.526 |
| | | | II | 2-Heptanol | alcohol | -0.586 |
| δ | | | III | 2-Nonanone | ketone | -0.419 |
| | | | III | 8-Nonen-2-one | ketone | -0.401 |
| | | *Vagococcus* | II | 2-Propanol | alcohol | -0.443 |
| | | | II | 2-Pentanol | alcohol | -0.390 |
| | | | II | 2-Heptanone | ketone | -0.547 |
| | | | II | Acetoin | ketone | -0.424 |
| | | | II | 2-Heptanol | alcohol | -0.529 |
| | | | III | 2-Nonanone | ketone | -0.434 |
| | | *Clostridiisalibacter* | II | 2-Propanol | alcohol | -0.366 |
| | | | II | 2-Pentanol | alcohol | -0.427 |
| | | | II | 2-Heptanone | ketone | -0.411 |
| | | | II | Acetoin | ketone | -0.485 |
| | *Proteobacteria* | *Marinobacter* | II | 2-Propanol | alcohol | -0.773 |
| | | | I | Ethanol | alcohol | -0.413 |
| | | | II | 2-Pentanone | ketone | -0.364 |
| | | | III | 2-Butanol | alcohol | -0.394 |
| | | | II | 2-Pentanol | alcohol | -0.762 |
| | | | II | 2-Heptanone | ketone | -0.560 |
| | | | II | Acetoin | ketone | -0.589 |
| | | | II | 2-Heptanol | alcohol | -0.638 |
| | | *Halomonas* | II | 2-Propanol | alcohol | -0.671 |
| | | | III | 2-Pentanone | ketone | -0.376 |
| | | | III | 2-Butanol | alcohol | -0.490 |
| | | | III | Methyl thiolacetate | sulfer | -0.429 |
| | | | II | 2-Pentanol | alcohol | -0.670 |
| | | | III | 3-Methylbutyl acetate | ester | -0.388 |
| | | | II | 2-Heptanone | ketone | -0.492 |
| | | | II | Acetoin | ketone | -0.470 |
| | | | II | 2-Heptanol | alcohol | -0.582 |
| | | | III | 2-Hydroxypentan-3-one | ketone | -0.454 |
| | | *Psychrobacter* | I | Propanal | aldehyde | -0.387 |
| | | | I | Ethyl Acetate | ester | -0.446 |
| | | | II | 2-Propanol | alcohol | -0.636 |
| | | | I | Ethanol | alcohol | -0.515 |
| | | | III | Methyl thiolacetate | sulfer | -0.413 |
| | | | I | Ethyl hexanoate | ester | -0.654 |
| | | | I | 3-Methylbutyl butanoate | ester | -0.394 |
| | | | II | Acetoin | ketone | -0.546 |
| | | | I | Propyl hexanoate | ester | -0.387 |
| | | | I | 3-Methylbutyl hexanoate | ester | -0.387 |

Table S12. Significant positive correlations between fungi and organic acids in cheese samples.

| Cluster (Fungi) | Genus | Cluster (Compounds) | Compound | Correlation (ρ) |
|---|---|---|---|---|
| β | *Debaryomyces* | I | Isobutyric acid | 0.403 |
| γ | *Kluyveromyces* | III | Formic acid | 0.365 |

Table S13. Significant negative correlations between fungi and organic acids in cheese samples.

| Cluster (Fungi) | Genus | Cluster (Compounds) | Compound | Correlation (ρ) |
|---|---|---|---|---|
| β | *Debaryomyces* | III | Formic acid | -0.370 |
| γ | *Penicillium* | I | Isobutyric acid | -0.400 |
| δ | *Dipodascus* | I | Isobutyric acid | -0.416 |
| | | II | 3-Methylbutanoic acid | -0.396 |

Table S14. Significant positive correlations between fungi and free amino acids in cheese samples.

| Cluster (Fungi) | Genus | Cluster (Compounds) | Compound | Correlation (ρ) |
|---|---|---|---|---|
| α | *Debaryomyces* | I | Lysine | 0.525 |
| β | *Penicillium* | II | Aspartic acid | 0.449 |
| γ | *Candida* | II | Arginine | 0.364 |

Table S15. Significant negative correlations between fungi and free amino acids in cheese samples.

| Cluster (Fungi) | Genus | Cluster (Compounds) | Compound | Correlation (ρ) |
|---|---|---|---|---|
| α | *Debaryomyces* | II | Aspartic acid | -0.401 |
| | | II | Proline | -0.362 |
| β | *Penicillium* | I | Tryptophan | -0.442 |
| | | I | Lysine | -0.473 |
| | *Pichia* | I | Lysine | -0.445 |
| γ | *Cyberlindnera* | I | Threonine | -0.544 |
| | | I | Serine | -0.511 |
| | | I | Alanine | -0.389 |
| | | I | Valine | -0.370 |
| | | I | Tyrosine | -0.499 |
| | | I | Lysine | -0.545 |
| | *Saturnispora* | I | Threonine | -0.463 |
| | | I | Serine | -0.480 |
| | | I | Histidine | -0.465 |
| | | I | Lysine | -0.465 |
| | *Kluyveromyces* | I | Threonine | -0.429 |
| | | I | Serine | -0.499 |
| | | I | Valine | -0.365 |
| | | I | Tyrosine | -0.371 |
| | | I | Lysine | -0.611 |
| | *Candida* | I | Threonine | -0.485 |
| | | I | Serine | -0.605 |
| | | I | Glutamic acid | -0.370 |
| | | I | Valine | -0.440 |
| | | I | Methionine | -0.499 |
| | | I | Isoleucine | -0.370 |
| | | I | Leucine | -0.385 |
| | | I | Tyrosine | -0.519 |
| | | I | GABA | -0.375 |
| | | I | Tryptophan | -0.475 |
| | | I | Lysine | -0.710 |

Table S16. Significant positive correlations between fungi and volatile compounds in cheese samples.

| Cluster (Fungi) | Genus | Cluster (Compounds) | Compound | Correlation (ρ) |
|---|---|---|---|---|
| α | *Dipodascus* | III | 2-Propanol | 0.539 |
| | | III | Methyl thiolacetate | 0.374 |
| | | II | 2-Pentanol | 0.449 |
| | | III | Acetoin | 0.576 |
| | | II | 2-Heptanol | 0.401 |
| | *Cyberlindnera* | II | 2-Pentanone | 0.560 |
| | | III | 2-Hexanone | 0.394 |
| | | II | 2-Pentanol | 0.582 |
| | | II | 2-Heptanone | 0.426 |
| | | II | 2-Heptanol | 0.544 |
| | *Pichia* | II | 2-Pentanone | 0.493 |
| | | II | 2-Pentanol | 0.500 |
| | | II | 2-Heptanone | 0.369 |
| | | II | 2-Heptanol | 0.414 |
| | *Saturnispora* | III | 2-Butanone | 0.371 |
| | | III | 2-Butanol | 0.476 |
| | | II | 2-Pentanol | 0.383 |
| | | III | Methyl 2-hydroxy-4-methylpemtanoate | 0.420 |
| | *Kluyveromyces* | III | 2-Propanol | 0.392 |
| | | II | 2-Pentanone | 0.496 |
| | | II | 2-Pentanol | 0.747 |
| | | II | 2-Heptanone | 0.422 |
| | | II | 2-Heptanol | 0.582 |
| | *Candida* | III | 2-Propanol | 0.369 |
| | | II | 2-Pentanone | 0.688 |
| | | III | 2-Butanol | 0.564 |
| | | III | Pentan-2-yl acetate | 0.363 |
| | | II | 2-Pentanol | 0.782 |
| | | III | 3-Methylbutyl acetate | 0.492 |
| | | II | 2-Heptanone | 0.490 |
| | | III | 2,4-Dithiapentane | 0.377 |
| | | II | 2-Heptanol | 0.598 |
| β | *Penicillium* | II | 2-Pentanone | 0.684 |
| | | II | 2-Pentanol | 0.554 |
| | | II | 2-Heptanone | 0.654 |
| | | II | 2-Heptanol | 0.461 |
| | | III | 2-Nonanone | 0.731 |
| | | III | 8-Nonen-2-one | 0.606 |
| | *Scopulariopsis* | III | 2-Hexanone | 0.483 |
| | | III | 2-Octanone | 0.474 |
| | | III | 2-Nonanone | 0.412 |
| | | III | 8-Nonen-2-one | 0.461 |
| | | III | 2-Decanone | 0.516 |
| | | III | 2-Nonanol | 0.363 |
| γ | *Debaryomyces* | I | 2,5-Dimethylpyrazine | 0.439 |
| | | I | 2,3,5-Trimethylpyrazine | 0.387 |

Table S17. Significant negative correlations between fungi and volatile compounds in cheese samples.

| Cluster (Fungi) | Genus | Cluster (Compounds) | Compound | Correlation (ρ) |
|---|---|---|---|---|
| | *Dipodascus* | III | 2-Butanone | -0.475 |
| | | I | 2,5-Dimethylpyrazine | -0.679 |
| | | I | 2,3,5-Trimethylpyrazine | -0.432 |
| | *Cyberlindnera* | I | Ethyl hexanoate | -0.412 |
| | | I | 2,5-Dimethylpyrazine | -0.458 |
| | *Pichia* | I | Ethyl hexanoate | -0.435 |
| α | | I | 2,5-Dimethylpyrazine | -0.363 |
| | *Kluyveromyces* | I | 2,5-Dimethylpyrazine | -0.634 |
| | *Candida* | I | Ethyl Acetate | -0.375 |
| | | I | Ethyl hexanoate | -0.479 |
| | | I | 3-Methylbutyl butanoate | -0.362 |
| | | I | 2,5-Dimethylpyrazine | -0.386 |
| | *Penicillium* | I | Ethyl Acetate | -0.377 |
| | | I | Ethyl hexanoate | -0.433 |
| | | I | 3-Methylbutyl butanoate | -0.454 |
| | | I | 2,3,5-Trimethylpyrazine | -0.424 |
| β | *Scopulariopsis* | I | 3-Methylbutanal | -0.632 |
| | | I | Dimethyl disulfide | -0.486 |
| | | I | 2-Methylpropan-1-ol | -0.430 |
| | | I | 3-Methyl-1-butanol | -0.522 |
| | | I | Ethyl hexanoate | -0.365 |
| | *Debaryomyces* | II | 2-Pentanone | -0.711 |
| | | II | 2-Pentanol | -0.775 |
| | | III | 3-Methylbutyl acetate | -0.411 |
| γ | | II | 2-Heptanone | -0.649 |
| | | II | 2-Heptanol | -0.648 |
| | | III | 2-Nonanone | -0.586 |
| | | III | 8-Nonen-2-one | -0.486 |
